# The human mitochondrial translation factor TACO1 alleviates mitoribosome stalling at polyproline stretches

**DOI:** 10.1093/nar/gkae645

**Published:** 2024-07-22

**Authors:** Michele Brischigliaro, Annika Krüger, J Conor Moran, Hana Antonicka, Ahram Ahn, Eric A Shoubridge, Joanna Rorbach, Antoni Barrientos

**Affiliations:** Department of Neurology, University of Miami Miller School of Medicine, 1600 NW 10^th^ Ave., Miami, FL 33136, USA; Department of Medical Biochemistry and Biophysics, Karolinska Institutet, Stockholm, Sweden; Max Planck Institute Biology of Ageing-Karolinska Institutet Laboratory, Karolinska Institutet, Stockholm, Sweden; Department of Biochemistry and Molecular Biology, University of Miami Miller School of Medicine, 1600 NW 10th Ave., Miami, FL 33136, USA; The University of Miami Medical Scientist Training Program (MSTP), 1600 NW 10th Ave.,Miami, FL33136, USA; The Neuro and Department of Human Genetics, McGill University, Montreal, QC, Canada; Department of Biochemistry and Molecular Biology, University of Miami Miller School of Medicine, 1600 NW 10th Ave., Miami, FL 33136, USA; The Neuro and Department of Human Genetics, McGill University, Montreal, QC, Canada; Department of Medical Biochemistry and Biophysics, Karolinska Institutet, Stockholm, Sweden; Max Planck Institute Biology of Ageing-Karolinska Institutet Laboratory, Karolinska Institutet, Stockholm, Sweden; Department of Neurology, University of Miami Miller School of Medicine, 1600 NW 10^th^ Ave., Miami, FL 33136, USA; Department of Biochemistry and Molecular Biology, University of Miami Miller School of Medicine, 1600 NW 10th Ave., Miami, FL 33136, USA; The Miami Veterans Affairs (VA) Medical System. 1201 NW 16th St, Miami, FL-33125, USA

## Abstract

The prokaryotic translation elongation factor P (EF-P) and the eukaryotic/archaeal counterparts eIF5A/aIF5A are proteins that serve a crucial role in mitigating ribosomal stalling during the translation of specific sequences, notably those containing consecutive proline residues (1,2). Although mitochondrial DNA-encoded proteins synthesized by mitochondrial ribosomes also contain polyproline stretches, an EF-P/eIF5A mitochondrial counterpart remains unidentified. Here, we show that the missing factor is TACO1, a protein causative of a juvenile form of neurodegenerative Leigh's syndrome associated with cytochrome *c* oxidase deficiency, until now believed to be a translational activator of *COX1* mRNA. By using a combination of metabolic labeling, puromycin release and mitoribosome profiling experiments, we show that TACO1 is required for the rapid synthesis of the polyproline-rich COX1 and COX3 cytochrome *c* oxidase subunits, while its requirement is negligible for other mitochondrial DNA-encoded proteins. In agreement with a role in translation efficiency regulation, we show that TACO1 cooperates with the N-terminal extension of the large ribosomal subunit bL27m to provide stability to the peptidyl-transferase center during elongation. This study illuminates the translation elongation dynamics within human mitochondria, a TACO1-mediated biological mechanism in place to mitigate mitoribosome stalling at polyproline stretches during protein synthesis, and the pathological implications of its malfunction.

## Graphical Abstract

**Graphical Abstract:**
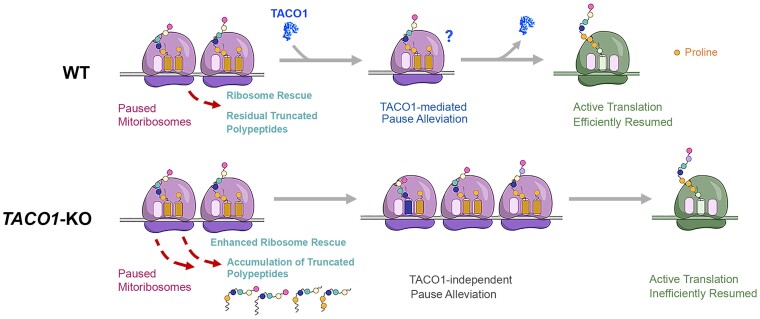

## Introduction

The human mitochondrial genome (mtDNA) encodes thirteen indispensable protein subunits of the oxidative phosphorylation (OXPHOS) enzymatic complexes that catalyze aerobic energy transduction, essential for sustaining life. Additionally, mtDNA encodes the *12S* and *16S* ribosomal rRNAs (rRNA) and 22 transfer RNAs (tRNAs) necessary for the synthesis of these proteins by mitochondrial ribosomes (mitoribosomes) (3). While significant strides have been made over the past decade in elucidating the structure, assembly, and function of mitoribosomes and the mechanisms governing translation (4–8), our understanding remains incomplete. Notably, while recent research has provided insights into the initiation and termination steps of mitochondrial translation (9–12), a substantial knowledge gap persists regarding factors regulating the elongation rate.

Extensive research into bacterial and cytosolic translation systems has revealed multiple mechanisms for regulating elongation speed (13,14). Firstly, variations in codon choice, context and distribution along the mRNA (15), and abundance of (aminoacyl-)tRNA influence the rates of substrate and tRNA selection and decoding, leading to ribosome pausing at rare codons or smooth progression with abundant codons/tRNAs. In mitochondria, the mtDNA comprises two strands of different compositions. The CA-rich light (L)-strand codes for 1 protein-coding gene (*ND6*) and 8 tRNA genes, and the GT-rich heavy (H)-strand codes for 12 protein-coding genes, 2 rRNA genes, and 14 tRNA genes. This strand-biased composition dictates codon usage in mitochondrial protein-coding sequences and the anticodon of tRNA genes (16,17). Secondly, mRNA secondary structures, such as specific stem-loops, can induce ribosomal pausing or stalling (18). In mitochondria, mRNA structures might also influence elongation rates, as exemplified by an mRNA-structure programmed translational pausing mechanism proposed to regulate the pace of cytochrome *c* oxidase subunit 1 (COX1) elongation and co-translational membrane insertion (19).

As a third mechanism, ribosome stalling can be triggered by polypeptide sequences, like those rich in lysine (Lys) and arginine (Arg), due to their strong electrostatic interactions with the negatively charged polypeptide exit tunnel of ribosomes (20). Furthermore, the amino acid proline (Pro) exhibits limited affinity as an acceptor in the A‐site during the peptidyl transferase reaction (21) and as a donor in the P-site (1). Consequently, consecutive proline residues can exert a pronounced inhibitory effect during elongation, leading to substantial ribosomal stalling observed in various bacterial, eukaryotic, and archaeal translation systems. In these systems, their translation machineries have recruited and selected auxiliary translation factors to accelerate the synthesis of polyproline stretches. Notably, prokaryotic elongation factor P (EF-P) and eukaryotic initiation factor 5A (eIF5A) play crucial roles in alleviating polyproline-induced ribosome stalling (1,2,22). Both EF-P and eIF5A occupy the E-site of the stalled ribosome and interact with the E-site surface of the P-site tRNA, providing stability, particularly to the CCA end of the P-site tRNA (23,24). Supporting their functional similarities and shared mechanisms of action, EF-P and eIF5A exhibit sequence and structural homology. EF-P adopts a three-domain architecture (25), while eIF5A is homologous to EF-P domains 1 and 2 but lacks the bacteria-specific domain 3 (26,27). Both proteins can undergo distinctive post-translational modifications (PTMs) on specific lysine residues at the tip of domain 1, which, when present, are crucial for their function, as they enable eIF5A or EF-P to reach the ribosome peptidyl-transferase center (PTC) (28,29). eIF5A is the only known protein to undergo hypusination (30). Differently, EF-P can undergo different modifications - β-lysinilation (31–33), arginine rhamnosylation (34,35) or 5-amino-pentanolylation (36–38) - depending on the bacterial phyla, or it may remain unmodified (39).

Proline-mediated helix kinks play a crucial role in facilitating the tight packing of transmembrane (TM) helices within membrane proteins (40). Despite the prevalence of polyproline stretches in the integral membrane proteins encoded by mtDNA, the precise mechanism through which the specialized mitochondrial protein synthesis machinery addresses this challenge remains elusive. Recent studies using an *in vitro* reconstituted system have uncovered that mitochondrial ribosomes are susceptible to polyproline-mediated ribosome arrest, a phenomenon distinct from those observed in reconstituted bacterial and cytosolic systems, as it is not alleviated by increasing the Mg^2+^ concentration in the reaction (41). However, a mitochondrial equivalent to EF-P/eIF5A has yet to be identified, representing a critical gap in our understanding of mitochondrial translation regulation.

Defects in mitochondrial translation represent a leading cause of mitochondrial diseases (5,42). In addition to classical translation initiation, elongation, and termination factors, only two proteins have been identified thus far that specifically influence the synthesis rate of particular polypeptides: leucine-rich pentatricopeptide repeat-containing protein (LRPPRC) (43) and the translational activator of the mtDNA-encoded cytochrome *c* oxidase subunit 1 (TACO1) (44,45). Mutations in the *LRPPRC* gene result in Leigh syndrome, French Canadian type, a devastating necrotizing infantile encephalopathy (46). While LRPPRC protein broadly impacts mt-mRNA stability and translation, excluding *ND6*, patients with LRPPRC mutations exhibit a distinct decrease in *COX1* and *COX3* mRNA levels and respiratory complex deficiencies that depend on the residual levels of LRPPRC found in tissues (47). For example, LRPPRC mutations lead to severe cytochrome *c* oxidase (COX) or respiratory chain complex IV (CIV) deficiency (46) and a secondary decrease in complex I in skeletal muscle, but milder CIV deficiency in heart muscle and secondary increases in CI and CIII levels (47). The *COX1* and *COX3* mRNAs are the most structured within the mitochondrial transcriptome (19), but they are also the least stable and poorly translated in the absence of LRPPRC (43,48,49). LRPPRC forms a complex with the SRA stem-loop-interacting RNA-binding protein (SLIRP), which directly interacts with all H-strand mRNAs (48,50) and facilitates their delivery to the mitoribosome during translation (43). On the other hand, mutations in TACO1 result in premature stop codons manifesting as a late-onset juvenile form of Leigh syndrome, also associated with COX deficiency (44,51,52). The primary molecular defect observed in affected individuals is a significant reduction in COX1 synthesis (44), a phenotype recapitulated in a mouse model (45). Initial studies of the *Saccharomyces cerevisiae* homolog (DPC29) did not identify any respiratory-deficient phenotype when the gene was ablated (44). However, more recent work on yeast suggested a more general, non-essential, and post-initiation translational control mediated by DPC29 (53). *In vitro* studies showed interactions of recombinant TACO1 with *COX1* mRNA and suggested that it is required for its translation by associating with the mitochondrial ribosome (45). Despite several efforts to elucidate the molecular function of TACO1 and the underlying mechanism of disorders arising from its loss, its precise role remains elusive.

Here, we have employed mitochondrial ribosome profiling and metabolic labeling of translation products in cells lacking TACO1 to demonstrate that TACO1 functions as a global mitochondrial translational elongation accelerator. Specifically, our data show that TACO1 is the translation factor responsible for mitigating polyproline-induced stalling of the human mitochondrial ribosome.

## Materials and methods

### Human cell lines and cell culture conditions

Human HEK293T embryonic kidney cells (CRL-3216, RRID: CVCL-0063) and 143B osteosarcoma cells (CRL-8303, RRID: CVCL-2270) were obtained from ATCC. Cybrid cell lines were constructed using enucleated control fibroblasts and the osteosarcoma 143B TK 206 rho zero cell lines (54). The *COX1* mutant cybrid cells carry a homoplasmic G6930A mitochondrial mutation that generates a stop at codon 542 and a truncated version of the COX1 protein (54). Cells were cultured in high-glucose Dulbecco's modified Eagle's medium (DMEM, Life Technologies) supplemented with 10% fetal bovine serum (FBS), 5 ml 1× antibiotic/antimycotic, 1 mM sodium pyruvate, and 50 μg/ml uridine, and 1× GlutaMAX (ThermoFisher Scientific) at 37°C under 5% CO_2_. Cell lines were routinely analyzed for mycoplasma contamination.

### Key reagents

Supplementary Table S1 includes the list of antibodies, recombinant DNAs, oligonucleotides, and siRNA oligoribonucleotides used in this study.

### Generation of knockout cell lines, plasmid transfection and selection

To engineer a *TACO1* knockout (KO) cell line, we obtained from Synthego Corporation (Redwood City, CA, USA) a pool of HEK293T cells that had been transfected with the gRNAs targeting *TACO1* exon 1. This pool was then plated into 96 well plates to screen for individual clones, and these clones were screened for the presence of TACO1 by immunoblotting. Clones that showed the absence of TACO1 were then genotyped by subcloning the edited genomic section into the pCR 2.1-TOPO TA vector. Clones were then picked, and the plasmid was sequenced by Sanger sequencing. To generate *TACO1*-KO cell lines reconstituted/rescued with untagged or tagged TACO1, a Myc-DDK-tagged *TACO1* ORF plasmid was obtained from OriGene Technologies (Rockville, MD, USA). This ORF was then subcloned into a hygromycin resistance-containing vector under the control of a full CMV6 promoter (pCMV6, OriGene) for overexpression or a Δ5-CMV6 truncated promoter (55) for achieving endogenous expression levels, using *Sfg*1 and *Mlu*1 sites. For the reconstitution of the *TACO1*-KO cell line with untagged *TACO1* ORF, we introduced a stop codon upstream of the Myc-DDK tag through Q5 Site-directed Mutagenesis Kit (New England Biolabs) according to the manufacturer's instructions. To create cell lines overexpressing bL27m, the wild-type *bL27m* ORF, obtained from OriGene, was subcloned in the hygromycin resistance-containing vector pCMV6 using the *Not*I site. bL27m mutants were generated with the Q5 Site-directed Mutagenesis Kit (New England Biolabs) according to the manufacturer's instructions. To transfect the plasmids into the corresponding cell lines, 0.3 × 10^6^ cells were seeded in 6-well plates. The next day, 1 μg of plasmid and 5 μl of EndoFectin (GeneCopoeia) were mixed with 125 μl of Opti-MEM reduced serum media (Gibco), respectively, and then combined. After a 20-min incubation, the DNA-reagent complexes were added to each well, and cells were selected after 48 h with media containing 200 μg/ml hygromycin B (Corning).

### Puromycin release experiment

For the puromycin release experiment, we pulse-labeled mitochondrial translation products with ^35^S-methionine in whole cells for increasing times (0–30 min) in the presence of emetine to inhibit cytoplasmic protein synthesis. Newly synthesized mitochondrial polypeptides were released by treating the cells with 4 μg/ml puromycin dihydrochloride from *Streptomyces alboniger* (Sigma).

### Denaturing and native electrophoresis followed by immunoblotting

For SDS-PAGE, cells were solubilized in RIPA buffer (50 mM Tris–HCl pH 8.0, 150 mM NaCl, 1% NP-40, 0.5% sodium deoxycholate, 2 mM EDTA and 0.1% SDS) with 1× EDTA-free protease inhibitor cocktail (Roche). Whole-cell extracts were cleared by 5 min centrifugation at 20 000 *× g* at 4°C. Equal amounts of total cellular protein were loaded in each lane and separated by SDS-PAGE on 12% or 14% polyacrylamide gels. Gels were transferred to a nitrocellulose membrane and then probed for immunoblotting with primary and secondary antibodies listed in Supplementary Table S1.

BN-PAGE analysis of mitochondrial respiratory chain complexes in native conditions was performed as described previously (56). Briefly, we pelleted and solubilized mitochondria in 100 μl buffer containing 1.5 M aminocaproic acid and 50 mM Bis–Tris (pH 7.0). Digitonin was added at 1:4 (digitonin: protein) proportion. Solubilized samples were incubated on ice for 5 min and pelleted at 20 000  ×  *g* for 30 min at 4 °C. The supernatant was supplemented with 10 μl of sample buffer 10X (750 mM aminocaproic acid, 50 mM Bis–Tris, 0.5 mM EDTA, 5% Serva Blue G-250) and loaded in 3–12% Native PAGE™ Bis–Tris Protein Gels (ThermoFisher Scientific). After electrophoresis, proteins were transferred to PVDF membranes using an eBlot L1 protein transfer system (GenScript, Piscataway, NJ) and probed for immunoblotting with primary and secondary antibodies listed in Supplementary Table S1.

### RNA extraction, reverse transcription and qRT-PCR

RNA was extracted from whole cells and sucrose gradient fractions using Trizol according to manufacturer's protocol. The aqueous phase was transferred to a new tube, and an equal volume of 100% isopropanol and 3 μl of 5 mg/ml glycogen were added to precipitate the RNA. The samples were incubated at -80 °C overnight and centrifuged at 15 000 ×*g* for 45 min, at 4°C. RNA was resuspended in RNAse-free water and quantified with Nanodrop (ThermoFisher Scientific). Reverse transcription was performed following the MIQE (minimum information for publication of quantitative real-time PCR experiments) guidelines (57) with the High-Capacity cDNA Reverse Transcription Kit (ThermoFisher Scientific). qRT-PCR was performed using SsoAdvanced Universal SYBR Green Supermix (Bio-Rad) and a Bio-Rad CFX 96 Touch System (Bio-Rad). The 2^–ΔΔCt^ method was used to assess the expression levels of the targets using beta-actin as endogenous control. The oligonucleotides used are listed in the Supplementary Table S1.

### Pulse labeling of mitochondrial translation products

To assess mitochondrial protein synthesis, 6-well plates were pre-coated at 5 μg/cm^2^ with 50 μg/ml collagen in 20 mM acetic acid and seeded with cells. Then, 70% confluent cell cultures were incubated for 30 min in DMEM without methionine and then supplemented with 100 μl/ml emetine for 10 min to inhibit cytosolic protein synthesis as described (58,59). 100 μCi of [^35^S]-methionine were added and allowed to incorporate into newly synthesized mitochondrial proteins for 30 min. Subsequently, whole-cell extracts were prepared by solubilization in RIPA buffer, and equal amounts of total cellular protein were loaded in each lane and separated by SDS-PAGE on a 17.5% polyacrylamide gel. Gels were transferred to a nitrocellulose membrane and exposed to a Kodak X-OMAT X-ray film. Membranes were subsequently probed with a primary antibody against β-ACTIN as a loading control.

### Mitochondria isolation

HEK293T cells were inoculated into one liter of Freestyle 293 expression medium supplemented with 50 ml FBS, 50 μg/ml uridine, and 10 ml of 100× antibiotic/antimycotic. Cells were grown at 37°C under 5% CO_2_, spinning at 63 RPM on an orbital shaker for five days. Cells were collected and resuspended in T–K–Mg buffer (10 mM Tris–HCl, pH 7.4, 10 mM KCl, 0.5 mM MgCl_2_) on ice and disrupted with 15 strokes in a pre-chilled homogenizer (Kimble/Kontes). A sucrose stock solution (1 M sucrose, 10 mM Tris–HCl, pH 7.4) was added to the homogenate to reach a final concentration of 0.25 M sucrose. A mitochondria-enriched supernatant was obtained by centrifuging the samples twice for 3 min at 1200 *× g* at 4°C. Mitochondria were pelleted by centrifugation for 10 min at 8000 *× g* and resuspended in 0.32 M sucrose, 10 mM Tris–HCl, pH 7.4, 1 mM EDTA.

### Sucrose gradient sedimentation analysis

Sucrose gradient sedimentation analyses were performed as described previously (60). Two mg of mitochondria were extracted in 400 μl of extraction buffer (20 mM HEPES pH 7.4, 100 mM KCl, 20 mM MgCl_2_, 0.60% digitonin, 0.5 mM PMSF, 1 × Protease Inhibitor, 40 U RNaseOUT). The lysate was ultracentrifuged at 24 000 *× g* for 15 min at 4°C. The supernatant was collected and loaded on top of a 5 ml 0.3 M to 1 M sucrose gradient solution (20 mM HEPES pH 7.4, 100 mM KCl, 20 mM MgCl_2_, 0.10% digitonin, 0.5 mM PMSF, 1 × protease inhibitor, 0.3 or 1 M sucrose, 0.5 mM ribonucleoside vanadyl complex). The gradients were centrifuged at 152 000 *× g* for 3 h and 10 min at 4°C, and then fractionated into 15 individual tubes, followed by SDS-PAGE and immunoblotting analysis.

To measure the relative abundance of *12S* and *16S* rRNAs in sucrose gradient fractions of a given gradient, the fraction containing the least RNA was used as the zero reference point to set the baseline for quantification, and the 2^–ΔCt^ method was used to assess the levels of the targets. Data smoothing was performed using B-spline interpolation (61) with a SciPy script. Data were analyzed in GraphPad Prism to generate the graphs.

### SILAC labeling and immunocapture

Wild-type and *TACO1*-FLAG expressing cell lines were grown in SILAC DMEM (Thermo Fisher) containing either ‘heavy’ ^15^N and ^13^C-labeled Arginine and Lysine (Sigma) or ‘light’ ^14^N- and ^12^C-Arginine and Lysine (Sigma) for at least 10 passages. Cells from 2 × 15 cm^2^ petri dishes per genotype were collected, and equal amounts of the differentially labeled cell lines were mixed. Mitochondria-enriched fractions were prepared by resuspending the cells in PBS and then treating the suspension with 2 mg/ml digitonin for 5 min on ice. Following two washes with cold PBS, these fractions were then solubilized in native conditions on ice for 10 min with PBS containing 10% glycerol, 1.5% DDM and 1× protease inhibitor cocktail (Roche). Insoluble material was pelleted by centrifugation at 20 000 ×*g* for 10 min at 4°C. Supernatants were collected and incubated with monoclonal anti-FLAG M2 affinity gel (Sigma), O/N at 4°C under gentle rotation. Beads were washed five times using PBS with 10% glycerol, 0.05% DDM and 1× protease inhibitor cocktail (Roche). Immunocaptured complexes were eluted under denaturing conditions using 1% SDS in PBS.

### Mass spectrometry

For MS analysis, samples in 40 μl of 1% SDS were supplemented with 12 μl of 20% SDS such that a final concentration of ∼5.4% SDS (v/v) was reached and processed for digestion using micro-S-Traps (Protifi) according to manufacturer instructions. Briefly, the samples were reduced with 2.26 μl of 0.5M dithiothreitol (DTT) at 56°C for 20 min, followed by alkylation using 2.26 μl of 0.55 M iodoacetamide (IAA) for 20 min at room temperature in the dark. An acidifier from Protifi (5.56 μl) was added, followed by 373 μl of Protifi's *buffer b*. Samples were then loaded onto the S-traps and washed according to the manufacturer's instructions. Finally, 8 μg of sequencing-grade trypsin (Promega) in 50 mM triethylammonium bicarbonate (TEAB) was added, and the mixture was incubated for 1 h at 47°C. Following this incubation, 40 μl of 50 mM TEAB was added to the S-Trap, and the peptides were eluted using centrifugation. Elution was repeated once. A third elution using 35 μl of 50% acetonitrile (ACN) was also performed, and the eluted peptides dried under a vacuum. Dried peptides were resolubilized in 50 μl of 1% trifluoroacetic acid (TFA) and desalted using 2 μg capacity ZipTips (Millipore, Billeric, MA) according to manufacturer instructions. Peptides were then on-line eluted into a Fusion Tribrid mass spectrometer (Thermo Fisher) from an EASY PepMap RSLC C18 column (2 μm, 100 Å, 75 μm × 50 cm, Thermo Fisher), using a gradient of 5–25% solvent B (80/20 acetonitrile/water, 0.1% formic acid) in 90 min, followed by 25–44% solvent B in 30 min, 44–80% solvent B in 0.1 min, a 10-min hold of 80% solvent B, a return to 5% solvent B in 3 min, and finally a 3-min hold of 5% solvent B. The gradient was then extended to clean the column by increasing solvent B to 98% in 3 min, a 98% solvent B hold for 10 min, a return to 5% solvent B in 3 min, a 5% solvent B hold for 3 min, an increase of solvent B again to 98% in 3 min, a 98% solvent B hold for 10min, a return to 5% solvent B in 3 min, a 5% solvent B hold for 3 min, and finally one last increase to 98% solvent B in 3 min and a 10-min hold at 98% solvent B. All flow rates were 250 nL/min delivered using a nEasy-LC1000 nano liquid chromatography system (Thermo Fisher). Solvent A consisted of water and 0.1% formic acid. Ions were created at 1.3 kV using an EASY Spray source (Thermo Fisher) held at 50°C. The Orbitrap Fusion Tribrid mass spectrometer was operated as previously described (62). The mass spectrometry analysis was performed at The Herbert Wertheim UF Scripps Institute for Biomedical Innovation & Technology, Mass Spectrometry and Proteomics Core Facility (RRID: SCR_023576).

### Proteomic data processing and statistical analysis

Quantitative analysis of the SILAC experiment was performed simultaneously to protein identification using Proteome Discoverer 2.5 (PD) software. The precursor and fragment ion mass tolerances were set to 10 ppm and 0.02 Da, respectively. The enzyme was Trypsin with a maximum of 2 missed cleavages and FASTA files for TACO1-FLAG protein, UP000005640 Human (downloaded on July 2023 with 20 813 entries; TACO1 protein was removed), and common contaminants were used in SEQUEST searches. The following settings were used to search the data; dynamic modifications; Oxidation/+15.995Da (M), Deamidated/+0.984 Da (N, Q), N-Terminal modification of Acetyl/+42.011 Da (N-Terminus), Met-loss/–131.040 Da (M), Met-loss + Acetyl/–89.030 Da (M), SILAC label: ^13^C(6)^15^N(2) of Lysine (+8.014Da), SILAC label:^13^C(6)^15^N(4) of Arginine (+10.008 Da) and static modification of Carbamidomethylation + 57.021 Da (C). Scaffold (version Scaffold_5.0.1, Proteome Software Inc.) was used to validate MS/MS-based peptide, protein identifications, quantification and statistical analysis. Both protein and peptide identifications were filtered with FDR < 1.0% by the Percolator posterior error probability calculation (63) for peptides and the Protein Prophet algorithm for proteins (64). In addition, proteins had to contain at least two identified peptides. Proteins that share similar peptides and could not be distinguished were placed in a protein group. Scaffold Q+ (version Scaffold_5.0.1, Proteome Software Inc.) was used to obtain quantitative values, and default Scaffold normalization was performed. Enriched proteins were determined by applying a *t*-test, further calculating FDR < 0.05 using the Benjamini–Hochberg method. The analysis was performed at The Herbert Wertheim UF Scripps Institute for Biomedical Innovation & Technology, Bioinformatics and Statistics Core Facility (RRID: SCR_023048). The MS hits list was filtered for mitochondrial proteins based on MitoCarta 3.0 (65). The filtered hits are listed in Supplementary Table S1.

### Ribosome profiling

Ribosome profiling (Ribo-Seq) was performed as described with some modifications (66).

#### Isolation and purification of mitoribosome-protected fragments (mtRPFs)

HEK293T WT, *TACO1*-KO and *TACO1*-KO + *TACO1* cells were grown to 80% confluency on 15-cm dishes. Medium was discarded and plates were shortly submerged in liquid nitrogen to snap freeze cells. 300 μl of 2× lysis buffer (100 mM Tris pH 7.5, 200 mM NaCl, 40 mM MgCl_2_, 2 mM DTT, 200 μg/ml chloramphenicol, 2% Triton X-100, 2× Complete EDTA-free protease inhibitor cocktail (Roche), 4000 U/ml TURBO DNase I (Thermo Fisher)) was added dropwise on each plate and lysates were collected using cell scrapers. Cell debris were removed by centrifugation (10 000 ×*g*, 15 min, 4°C). 200 μl of supernatant were subjected to RNase treatment for 30 min at RT (350 U, Ambion RNase I, Thermo Fisher). RNase treatment was stopped by the addition of 15 μl RNase inhibitor (1 U/μl, SUPERase-In, Thermo Fisher), which was followed by a short centrifugation step to remove insoluble material (5000 ×*g*, 5 min). Supernatants were loaded on 10–30% sucrose gradients (50 mM Tris pH 7.4, 100 mM NaCl, 20 mM MgCl_2_, 1 mM DTT, 100 μg/ml chloramphenicol, 1× Complete EDTA-free protease inhibitor cocktail (Roche), 40 U/ml RNase inhibitor (SUPERase-In, Thermo Fisher) in 11 × 34 mm tubes (Beckman Coulter) and run at 39 000 rpm for 2 h 15 min in a TLS-55 rotor (Beckman Coulter). Afterward, 100 μl fractions were collected, and 10 μl of each was used for immunoblotting analysis. The remaining volumes of fractions 11–16, representing monosome-containing fractions, were combined and further processed for mitoribosome profiling. First, RNA was isolated from pooled fractions using TRIZOL reagent (Thermo Fisher) according to the manufacturer's instructions. Isolated RNA was heated at 80°C for 3 min, put on ice for 1 min, mixed with Gel Loading Buffer II (Thermo Fisher), and loaded onto a 15% Novex TBE–urea gel (Thermo Fisher). The gel was run in 1× TBE buffer at 100 V for ∼2 h. After completion of the run, the gel was stained with 1× SYBR Gold Nucleic Acid Gel Stain (Thermo Fisher) in 1× TBE. Nucleic acids were visualized, and bands sizing from 30 to 40 nt were excised. RNA was extracted from gel slices in 600 μl RNA extraction buffer (300 mM NaOAc pH 5.5, 1 mM EDTA, 0.25% SDS) rotating at 4°C ON. The next day, RNA was precipitated by adding 1.8 ml ice-cold EtOH and 4 μl GlycoBlue Coprecipitant (Thermo Fisher) and subsequent storage at –80°C ON. Precipitated RNA was pelleted by centrifugation (5000 ×*g*, 10 min, 4°C). Pellet was once washed with 1 ml EtOH, dried for ∼5 min, and resuspended in 15 μl 10 mM Tris pH 7.5 supplemented with 1 μl RNase inhibitor (SUPERase-In, Thermo Fisher).

#### Ligation of adaptors to mtRPFs

Samples were heated at 80°C for 2 min before placing on ice. Next, 3′ phosphates were removed by T4 PNK treatment (1 μl T4 PNK (NEB) added) in 1× T4 PNK buffer (NEB) at 37°C for 2 h. The reaction was stopped by heat inactivation at 65°C for 10 min. RNA was pelleted by the addition of 70 μl water, 2 μl GlycoBlue Coprecipitant (Thermo Fisher), 10 μl 1 M NaOAc, and 300 μl EtOH and subsequent storage at –80°C. RNA was washed and dried as described earlier and finally resuspended in 7 μl 10 mM Tris pH 7.5 supplemented with 1 μl RNase inhibitor. RNA libraries were generated using TrueSeq Small RNA Library Prep Kit (Illumina) according to the manufacturer's protocol with some modifications. Preparation was started by adding 1.2 μl adenylated RA3 to dephosphorylated RNA and incubating the mixture at 80°C for 2 min. Afterward, ligation was performed by the addition of 2 μl of T4 RNA Ligase 2 (truncated K227Q), 2 μl T4 RNA Ligase 2 buffer, and 6 μl PEG8000 (all components from NEB) and incubation at 14°C ON. RNA was precipitated as described earlier, 20 μl 3 M NaOAc and 600 μl EtOH) and resuspended in 4 μl 10 mM Tris pH 7.5. Ligation products were then purified on a 15% Novex TBE–urea gel (Thermo Fisher), extracted, and precipitated as described earlier. Next, RNA was resuspended in 13 μl 10 mM Tris pH 7.5 supplemented with 1 μl RNase inhibitor. Then, 2 mM ATP, 2 μl 10× T4 PNK buffer and 2 μl T4 PNK (NEB) were added, and the reaction mixture was incubated for 2 h at 37°C, followed by heat inactivation (65°C, 10 min). RNA was precipitated and resuspended in 13 μl 10 mM Tris pH 7.5 supplemented with 1 μl RNase inhibitor. Thereafter, RNA footprints were ligated with a 5′ RNA adaptor (RA5, Illumina) by adding 1.2 μl RA5, 2 μl 10× T4 buffer, and 2 μl T4 RNA ligase (Promega) and incubating at 14°C ON. RNA was precipitated and resuspended in 3 μl 10 mM Tris pH 7.5.

#### Reverse transcription and PCR amplification of library

Reverse transcription was performed using RNA RT primers from TrueSeq Small RNA Library Prep Kit (Illumina) and SuperScript III First-Strand Synthesis System (Thermo Fisher) according to the manufacturer's protocol. Afterward, 2 μl of RT products were PCR amplified using Phusion High-Fidelity PCR master mix (NEB) and DNA primers from TrueSeq Small RNA Library Prep Kit (Illumina). The PCR products were resolved on a 10% Novex non-denaturing TBE gel (Thermo Fisher) using 1× TBE running buffer. PCR products were excised and extracted using DNA extraction buffer (300 mM NaCl, 10 mM Tris pH 8, 1 mM EDTA). Subsequently, PCR products were precipitated and pelleted. Libraries were resuspended in12 μl 10 mM Tris pH 7.5.

#### Duplex-specific nuclease (DSN) digestion

To reduce the amount of ribosomal RNA contamination, DSN digestion was performed using a DSN kit (Evrogen). First, the libraries were added 4 μl of hybridization buffer (200 mM HEPES pH 7.5, 2 M NaCl). Next, libraries were heated for 2 min at 98°C and incubated for 5 h at 68°C. Consecutively, 1x master buffer (Evrogen) and 2 μl DSN enzyme were added to the samples and incubated for 25 min at 68°C. Digestion was stopped by adding 20 μl stop solution (Evrogen) and 5 min incubation at 68°C. Finally, samples were cooled down on ice, and DNA was isolated using phenol/chloroform extraction. Therefore, samples were mixed with 160 μl water and 200 μl phenol/chloroform (1:1), and the aqueous phase was precipitated as before. 2 μl of digested libraries were subjected to another round of PCR amplification and consecutive gel purification. Final libraries were resuspended in 11 μl 10 mM Tris pH 7.5. Fragment size distribution of the sequencing libraries was assessed by gel electrophoresis using Agilent's Bioanalyzer High Sensitivity dsDNA kit, and final library concentrations were quantified using Thermofisher's Qubit 1x dsDNA high sensitivity kit. The libraries were then pooled in equimolar amounts and paired-end sequencing was performed on a Nextseq550 to obtain ∼20 million reads per sample (settings: read 1 = 75 cycles, index 1 = 6 cycles, read 2 = 75 cycles).

#### Computational analysis of mRPFs

The pooled sequencing data were split by the index barcodes used for each sample during the pooling PCR and converted to the FASTAQ format by bcl2fastq (Illumina). First, the common 3′ and 5′ adapter sequences were trimmed off from the adaptor reads by using Cutadapt with parameters: ‘-a TGGAATTCTCGGGTGCCAAGG -A GATCGTCGGACTGTAGAACTCT-GAAC’ and read lengths were filtered with parameters: ‘-m 25 -M 45’ (67). Then, the trimmed reads were aligned to the mitochondrial transcriptome using Bowtie2 local alignment (68). The A-site of the mapped reads was identified using a plastid (69). First, P-site offsets were determined based on stop codon peaks of COX1, followed by adjustments via read-phasing analysis. Final offsets were set between 15–18 nucleotides. These offsets were used to calculate mitoribosome counts per transcript and generate count vectors. Further analysis was performed in RStudio 2021.09.0 + 351 using custom-made scripts. To analyze relative ribosome distributions on transcripts, read counts were normalized to the total number of reads on all mitochondrial transcripts. To analyze codon-specific ribosome positions on individual transcripts, read counts on each codon position were normalized to the total number of reads on the respective transcript. Occupancy of ribosomes in proline-rich regions was determined as follows: A region length of 5 amino acids was chosen, and each transcript was scanned for the number of prolines within this region length by computing rolling sums. Next, regions were grouped according to the number of prolines, and the respective relative occupancies of ribosomes in these regions were summed. Finally, the relative occupancy of ribosomes in the different proline-rich regions was normalized to WT.

### BioID proximity labeling

BioID analysis of TACO1 protein was performed as part of a larger group of 100 mitochondrial baits (14). Specifically, the BirA*-FLAG construct was generated using Gateway cloning into the pDEST5-BirA*FLAG-C-ter vector with TACO1 lacking a stop codon in an entry clone V120806 (Lunenfeld-Tanenbaum Research Institute Open Freezer (Toronto, Canada). Flp-In T-REx 293 cells expressing TACO1-BirA* were created as described previously (14). Purification of biotinylated proteins, followed by their identification by mass spectrometry, was performed in biological duplicates (as defined by two separate harvests) described previously (70), and data were compared to negative controls expressing the BirA* tag fused to GFP, and untransfected cells using SAINTexpress with default parameters (71). Forty-eight independent negative controls were performed; to increase the stringency in scoring, the 24 maximal spectral counts across the 48 controls were used for each prey protein, generating 24 ‘virtual controls’ against which the TACO1 results were scored. All preys detected with TACO1 that passed a Bayesian False Discovery Rare (BFDR) cutoff of ≤0.01 are listed in Supplementary Table S3. The prey specificity module of the ProHits-viz software (prohits-viz.org) was used to score specific prey enrichment with TACO1 against all other mitochondrial baits profiled, using spectral counts as a proxy for relative abundance. GO term analysis was performed using PANTHER Overrepresentation Test (pantherdb.org; Released 20190711).

### Statistical analysis

All the experiments were done in triplicate or otherwise indicated in the figure legends. Data in X-ray films were digitalized and analyzed using the ImageJ software or the Adobe Photoshop histogram option. Statistical analyses were performed using GraphPad Prism Software, version 8.2.1, and Microsoft Excel. The details of the software used in this study are listed in Supplementary Table S1. The data are presented as the means ± S.D. or mean ± S.E.M. of absolute values or percentages of control. The values obtained for transfected and non-transfected WT and the *TACO1*-KO cell lines for the different parameters studied were compared using a Student's two-tailed unpaired *t*-test for comparison of two groups. For comparison of multiple groups, we performed a two-way analysis of variance (ANOVA) followed in most cases by a Dunnett's multiple comparisons test: (*P < 0.05; **P< 0.01; ***P < 0.001; ***P < 0.0001). Sample numbers, statistical tests, and statistical significance are described in the figure captions.

## Results

### The absence of TACO1 leads to decreased COX1 and COX3 synthesis and accumulation of truncated de novo synthesized polypeptides.

**Figure 1.**
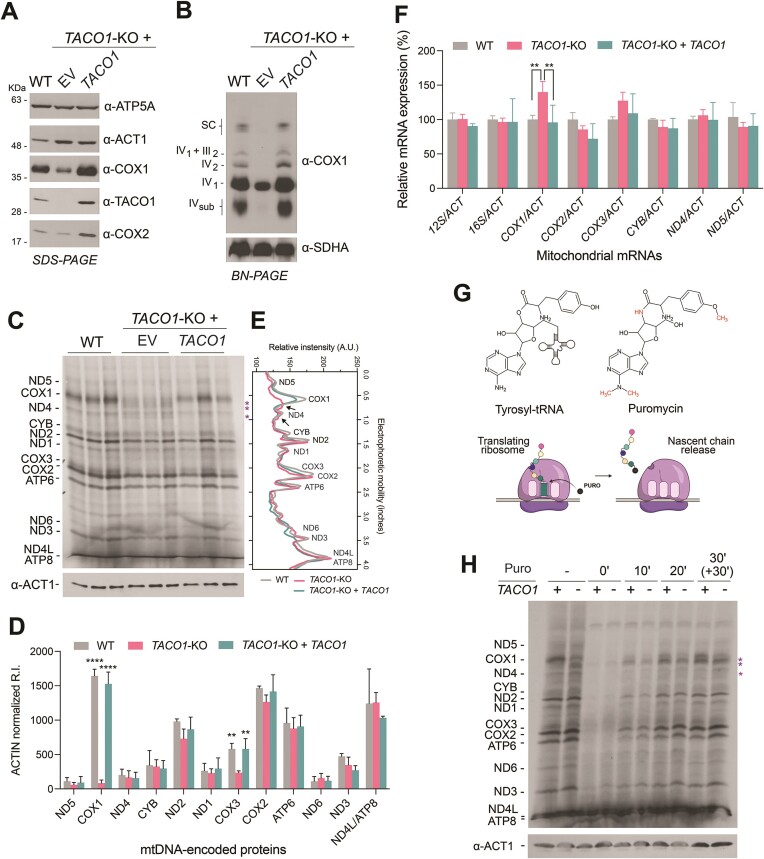
Loss of TACO1 causes cytochrome *c* oxidase deficiency due to aberrant translation of *COX1* mRNA. (**A**) Steady-state levels of TACO1, cytochrome *c* oxidase subunits (COX1, COX2), F_1_F_o_-ATP synthase subunit ATP5A, and β-ACTIN in WT, *TACO1*-KO and *TACO1*-KO rescued HEK293T cells. (**B**) BN-PAGE analysis of MRC complexes IV and II in WT, *TACO1*-KO and *TACO1*-KO rescued HEK293T cells. (**C**) Metabolic labeling of newly synthesized mitochondrial translation products with ^35^S-methionine in the presence of emetine to inhibit cytoplasmic protein synthesis in WT, *TACO1*-KO and *TACO1*-KO rescued HEK293T cells. Aberrant products of mitochondrial translation observed in *TACO1*-KO cells are highlighted with asterisks. Immunoblot analysis against β-ACTIN is provided as a loading control. (**D**) Densitometric quantification of the newly synthesized mtDNA-encoded proteins. Data are plotted as mean ± SD (*n* = 3 biological replicates, two-way ANOVA with Dunnet's multiple comparisons, ****P ≤ 0.0001, **P ≤ 0.01). (**E**) Distribution profiles of ^35^S-labeled products of mitochondrial protein synthesis are shown in (C). Profiles show the average distribution of the triplicate per each genotype. (**F**) Transcript levels of mtDNA-encoded genes, normalized by *ACTIN* mRNA (*ACT*) levels. Data are plotted as mean ± SD (*n* = 3 biological replicates, two-way ANOVA with Sidak's multiple comparisons, **P ≤ 0.01). (**G**) Schematic representation of the puromycin mechanism of action. The top panels show the chemical structures of the tyrosyl-tRNA end and puromycin. The bottom panels illustrate that during protein synthesis, puromycin gets incorporated in an unspecific amino-acid manner and causes translation termination with the release of the nascent polypeptide chain. (**H**) Puromycin release experiment. Labeling of mitochondrial translation polypeptides with ^35^S-methionine in WT and *TACO1*-KO cells as in Figure 1C but for each cell line, in the absence (–) or presence of puromycin during the translation assay. Puromycin was added at the beginning of the ^35^S-methionine labeling (0′) or after increasing times of pulse-labeling (10′, 20′ and 30′). The 35S-methionine pulse-labeling was extended in an additional sample for thirty additional minutes (+30′). The main puromycin-released translation products are highlighted with an asterisk. Immunoblot analysis against β-ACTIN is provided as a loading control.

To investigate the role of TACO1 on mitochondrial translation, we used the CRISPR-Cas9 gene editing system to generate a human HEK293T cell line knockout (KO) for TACO1 (*TACO1*-KO). Consistent with previous findings in *TACO1* patient fibroblasts (44) and *TACO1*-deficient mouse tissues (45), *TACO1*-KO cells exhibited decreased steady-state levels of COX1 and COX2 subunits compared with wild-type (WT) cells (Figure 1A). Consequently, *TACO1*-KO cells had reduced levels of fully assembled monomeric and dimeric forms of respiratory chain CIV as well as CIV-containing respiratory supercomplexes (III_2_+ IV and I + III_2_+ IV SC) (Figure 1B). Metabolic labeling of mitochondrial translation products with ^35^S-methionine revealed a severe reduction in COX1 synthesis (approximately 10% residual full-length COX1 synthesis) (Figure 1C, D) accompanied by a less pronounced decrease in COX3 synthesis (Figure 1C, D), as seen in *TACO1* patient fibroblasts (44). Notably, our metabolic labeling also revealed the presence of at least three novel aberrant mitochondrial translation products, exclusively detected in *TACO1*-KO cells, migrating just below COX1 in the SDS-PAGE system (Figure 1C, E). To exclude that these phenotypes could stem from altered mitochondrial transcription or RNA stability, we subsequently analyzed the abundance of several mitochondrial RNAs, including *COX1, COX2, COX3, CYB, ND4* and *ND5* mRNAs, as well as the *12S* and *16S* rRNAs. While most transcripts showed comparable levels between WT and KO cells, we observed a statistically significant 1.5-fold increase in *COX1* levels and a non-significant trend towards elevated *COX3* levels in *TACO1*-KO cells (Figure 1F). Given the polycistronic nature of mitochondrial transcription, our data suggest enhanced stability of poorly translated transcripts in the absence of TACO1. Crucially, all observed phenotypes in *TACO1*-KO cells were rescued upon expression of plasmid-borne recombinant *TACO1*, thereby eliminating the possibility of significant off-target effects resulting from the CRISPR-Cas9-mediated gene editing (Figure 1A–F).

### The truncated newly-synthesized polypeptides are early-released COX1 fragments.

Considering the prominent COX1 translation defect observed in both patients and model systems lacking TACO1, we aimed to investigate whether the abnormal mitochondrial translation products detected in *TACO1*-KO cells are truncated products resulting from interrupted *COX1* mRNA translation. To test this hypothesis, we knocked out *TACO1* in WT 143B cells and in 143B cybrids carrying a *COX1* homoplasmic mutation that induces a premature stop at codon 342 (54). WT 143B cells lacking TACO1 showed a profound decrease in COX1 steady-state levels (Supplementary Figure S1A) and a pattern of de novo translation products (Supplementary Figure S1B) similar to the pattern observed in HEK293T cells (Figure 1C). COX1 expression in the cybrids was virtually absent, regardless of the presence of TACO1 (Supplementary Figure S1A). However, metabolic labeling of mitochondrial protein synthesis in the *TACO1*-KO cybrids failed to detect the additional polypeptides migrating under COX1 observed in *TACO1*-KO carrying a WT *COX1* gene (Supplementary Fig. S1B). These results demonstrate that the truncated products observed arise during COX1 synthesis and strongly suggest that they originate from premature termination events occurring downstream of the mutation-induced stop at codon 342.

Having established the source of the abnormal translation products caused by the TACO1 mutation, we used the protein synthesis experiment conducted on HEK293T cells (Figure 1C) to quantitatively assess the products derived from *COX1* mRNA translation. Our data revealed comparable levels of COX1 synthesized in WT cells and the combined levels of COX1 and COX1-truncated polypeptides in *TACO1*-KO cells (Supplementary Figure S1C). This finding supports the notion that the COX1 translational defect induced by the loss of TACO1 does not stem from inefficient translation initiation/loading of the *COX1* mRNA onto the mitoribosome but rather occurs during elongation due to premature termination events.

To gain further insight into the nature of the truncated COX1 polypeptides, we conducted a puromycin release experiment. Puromycin, acting as a tyrosyl tRNA analog, incorporates into the nascent polypeptide chain during translation, leading to termination and subsequent release of the nascent chain (72) (Figure 1G). In a pulse-labeling protein synthesis experiment conducted on both wild-type (WT) and *TACO1*-KO HEK293T cells, puromycin was added at time 0 and increasing intervals during the labeling period. Unexpectedly, we observed prominent nascent chains released in WT cells under all tested conditions, displaying the same electrophoretic mobility as the COX1 fragments generated due to the loss of TACO1 (Figure 1H).

From these results, we deduce that ribosome stalling during *COX1* mRNA translation occurs at specific sequences regardless of the presence or absence of TACO1. However, while WT cells can efficiently resolve the pause, resume translation, and complete COX1 synthesis, this process is notably inefficient without TACO1.

### TACO1 transiently interacts with translating mitoribosomes

Previous work has suggested that TACO1 transiently associates with the mitoribosome (45). To validate such interaction, we used several approaches. First, we performed sucrose gradient sedimentation analysis of HEK293T mitochondrial extracts prepared in the presence of 0.6% digitonin, 100 mM KCl, and 20 mM MgCl_2_ to preserve mitoribosome subunit interactions within monosomes. The sedimentation pattern of TACO1 in our high-resolution gradients showed that a small portion (<5%) of the protein co-sediments with a mitoribosome large subunit (mtLSU) marker (uL14m) in the mtLSU, monosome, and polysome-containing fractions (Supplementary Figure S2A). In contrast, the bulk of TACO1 accumulated in lighter fractions, either alone or as part of a small complex. To assess the effect of excess TACO1, we engineered WT and *TACO1*-KO cell lines wherein recombinant *TACO1* expression was governed by a full-length CMV6 promoter. In these cell lines, the steady-state levels of TACO1 were elevated by 5–10-fold compared to endogenous WT levels (Supplementary Figures S2B and S2C), and the fraction of TACO1 co-sedimenting with the mitoribosomes, particularly with the mtLSU particle, was slightly enhanced (Supplementary Figure S2D).

**Figure 2.**
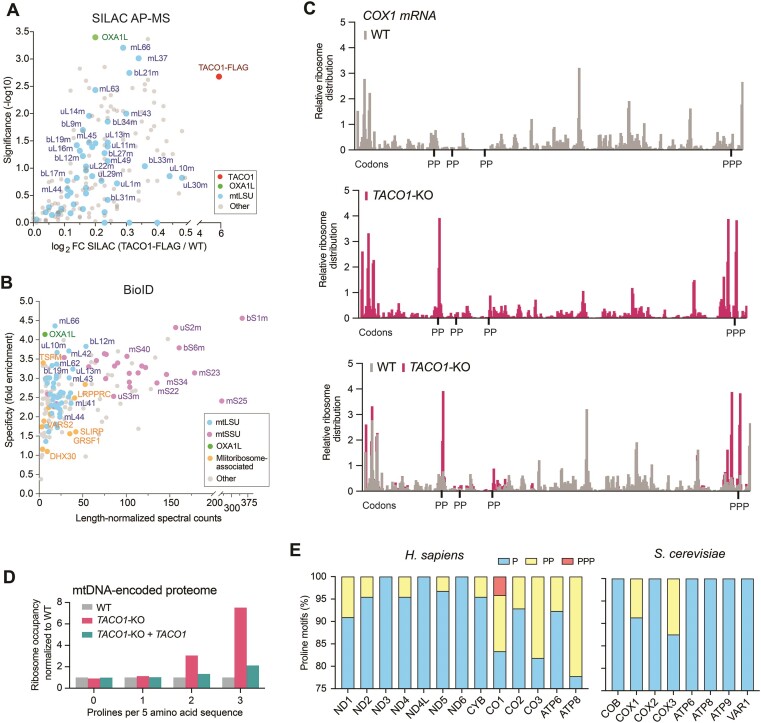
TACO1 binds to the mitoribosome and positively regulates mitochondrial protein synthesis by alleviating polyproline-induced mitoribosome stalling. (**A**) SILAC-based quantitative proteomic analysis of TACO1 interactors in native conditions. Enriched proteins were determined by applying a t-test, further calculating FDR < 0.05 using Benjamini-Hochberg correction. Red = TACO1-FLAG (bait) is marked in red. Enriched mtLSU ribosome proteins and the OXA1L insertase are highlighted in blue and green colors, respectively. (**B**) Prey specificity graph for BioID proximity interactome of TACO1 protein, where prey specificity was determined as the relative enrichment of interaction of individual preys and TACO1, compared to their interaction with 100 other mitochondrial baits. Enriched mtLSU ribosome proteins and the OXA1L insertase are highlighted in the same colors as in panel (A). mtSSU proteins and mitoribosome-associated proteins are color-coded in purple and orange, respectively. (**C**) Relative occupancy of mitoribosomes on COX1 transcript in WT and *TACO1*-KO cells analyzed by ribosome profiling experiments. Read counts at each codon position were normalized to the total number of read counts for COX1. Proline-rich regions of more than two prolines per 5 amino acid sequence length are highlighted. (**D**) Relative ribosome occupancies in regions having 0–3 prolines per 5 amino acid sequence length. Ribosome occupancies in *TACO1*-KO and *TACO1* reconstituted cells were normalized to WT cells. (**E**) Relative distribution of polyproline motifs in human and yeast mtDNA-encoded proteins.

To identify proteins directly interacting with TACO1 or constituting TACO1-containing protein complexes, we employed stable isotope labeling of amino acids in cell culture (SILAC) (73). This involved using WT cells and *TACO1*-KO cells expressing functional TACO1-FLAG to conduct FLAG-affinity purification under native conditions, followed by quantitative mass spectrometry (AP-MS). Through this methodology, we observed the co-immunopurification of several mtLSU proteins but none from the mitoribosome small subunit (mtSSU) with the highly enriched TACO1-FLAG (Figure 2A and Supplementary Table S2), corroborating findings from sucrose sedimentation assays. Remarkably, the OXA1L insertase, known for its association with the translating mitoribosomes to aid in the membrane insertion of newly synthesized mtDNA-encoded proteins (74), was also co-immunoprecipitated, suggesting that the association of TACO1 with mitoribosomes occurs during active translation. The absence of mtSSU proteins in the immunoprecipitate can be explained by the preferential association of TACO1 with the mtLSU, as well as the dissociation of the monosome during the extraction and affinity purification procedures.

As an alternative strategy to unravel protein associations with TACO1, we employed the proximity-biotinylation assay (BioID), a method enabling the detection of nearby protein partners (∼10 nm radius) within living cells (75,76). As part of a broader investigation, we engineered a HEK293 Flp-In T-REx cell line expressing the TACO1-BirA* fusion protein from an inducible construct stably integrated into the genome. The mitochondrial localization of the fusion protein was confirmed via immunofluorescence microscopy (76). BioID experiments were conducted in biological replicates, and the resulting mass spectrometry data was analyzed against a set of negative controls consisting of cells expressing the BirA* fused to GFP or untransfected cells. This analysis yielded a catalog of high-confidence proximity partners for TACO1 (Supplementary Table S3). Gene Ontology (GO) term analysis revealed an enrichment of mitoribosome components and matrix proteins, particularly those involved in mitochondrial translation. To define whether any of these proteins were specifically enriched when TACO1 was used as bait, we took advantage of the large dataset of ∼100 mitochondrial baits (76) and performed specificity enrichment analysis of the baits in proximity of TACO1. The specificity enrichment analysis calculates the fold enrichment of the proximity interaction (based on the detected spectral counts) for each prey for the bait of interest (TACO1) and compares it to all the other baits in the dataset. Setting a length-normalized spectral counts limit to a minimum of 2, the specificity plot of preys detected with TACO1-BirA* highlights mtLSU and mtSSU mitoribosomal proteins and OXA1L as the most specific proximity interactors (Figure 2B), aligning with the SILAC AP-MS studies (Figure 2A). The data from this assay indicate that TACO1 is in proximity to the whole mitochondrial monosome, as subunits of both mtLSU and mtSSU are biotinylated. Although proximity labeling does not necessarily indicate a direct interaction, we observed that the five mtLSU prey proteins with the highest specificity scores (ml66, bL12m, uL10m, mL42 and mL62) are located by the L7/L12 stalk near the tRNA-acceptor site (A-site) (Supplementary Fig. S4). In contrast, uL1m, which is closer to the tRNA-exit site (E-site), had a medium-low specificity score, ranking in the middle of the mtLSU preys (Supplementary Table S3). As a reference, the BioID specificity data for the mitochondrial elongation factor GFM1 identified uL10m as the top mtLSU prey (76).

In conclusion, the congruent results across the different approaches indicate that a minute fraction of TACO1 associates with the mtLSU and mitoribosomes during active translation.

### Synthesis of all mitochondrial polypeptides, most notably COX1 and COX3, is stalled at polyproline stretches

To identify the entire translation landscape under the control of TACO1, we used mitoribosome profiling, an approach that provides direct, global, and quantitative measurements of mitochondrial protein synthesis rates.

First, we analyzed the overall distribution of mitoribosomes on mitochondrial transcripts in WT, *TACO1*-KO and *TACO1* reconstituted cells. Mitoribosome occupancies were similar between the three tested cell lines (Supplementary Figure S3A), supporting our previous observations that decreased COX1 and COX3 de novo protein levels in *TACO1*-KO cells were not caused by transcript-specific failures in translation initiation events (Figure 1C). To investigate mitoribosome elongation rates on different transcripts, we calculated mitoribosome positions with single codon resolution. Comparing mitoribosome occupancies between WT and *TACO1*-KO cells, we identified 13 stalling events across the mtDNA-encoded proteome that were alleviated in WT cells by the presence of TACO1 (Figure 2C, Supplementary Figure S3C and Supplementary Tables S4–S6). Importantly, these stalling events observed in the *TACO1*-KO cell line were rescued by the reintroduction of *TACO1* (Supplementary Figure S3B). Of the TACO1-associated 13 stalling events, 3 occurred in COX1, 2 in COX3 and ATP6, and 1 in each COX2, ND2, ND3, ND4L, ND6 and ATP8 (Figure 2C, Supplementary Fig. S3C and Supplementary Tables S4–S6). Of the nine proteins whose synthesis efficiency is regulated by TACO1, only pauses during translation of *COX1* and, to a minor extent, *COX3*, were associated with a noticeable effect in protein synthesis rate in the *TACO1*-KO cell line (Figure 1). Analysis of the three stalling sites in the *COX1* transcript revealed that they are associated with polyprolines, with two featuring a 2xPro sequence, and the third consisting of a 3xPro motif (Figure 2C, Supplementary Table S4). Proline causes translating ribosomes to pause because their pyrrolidine ring confers exceptional conformational rigidity compared to all other amino acids, rendering it not only a poor A-site peptidyl acceptor but also a poor P-site peptidyl donor (21). As ribosome stalling is known to induce ribosome rescue mechanisms leading to non-canonical translation termination events (77), it is reasonable to hypothesize that the observed truncated COX1 proteins detected in *TACO1*-KO cells (Figure 1) are caused by pre-termination at the 3xPro motif, which is located close to the end of *COX1* ORF (Figure 2B). In addition to the pause at the PPP triplet, two other distinct pauses were observed 10 aa and 16 aa upstream the central P of the triplet, at the underlined aa in the sequence KVLMVEEPSMNLEWLYGCPPP. Given the mitoribosome footprint length of 32 nt with the P-site position in the footprints being 12 nt downstream from the 5′ ends (78), the premature termination at the two sites upstream of the 3xPro motif may result from mitoribosome rescue mechanisms triggered by collided mitodisomes, as previously shown in bacteria (79). Therefore, the pronounced stalling of mitoribosomes at the COX1 3xPro motif in *TACO1*-KO cells clarifies why the translation of *COX1* is profoundly impacted by the absence of TACO1 in humans (44).

To test if the absence of TACO1 induces a general stalling of mitoribosomes at proline motifs, we compared mitoribosome occupancies at proline-rich sequence stretches between the different cell lines (Figure 2D). Indeed, we observed an accumulation of mitoribosomes in regions harboring multiple prolines in the absence of TACO1. Upon examining the distribution of 1xPro, 2xPro and 3xPro sequences within human mitochondrial transcripts (Figure 2E, and Supplementary data SD1), we observed that all mtDNA-encoded proteins, except ND3, ND4L, and ND6, contain at least one 2xPro sequence, and that COX1 has the single 3xPro motif in the mtDNA-encoded proteome.

In contrast to humans, the loss of function of DPC29, the yeast homolog of TACO1, results in an extremely subtle phenotype (53). This is consistent with the observation that yeast mtDNA-encoded proteins have fewer polyproline motifs than their human counterparts, with only COX1 and COX3 containing 2xPro motifs (Figure 2E). Interestingly, mtDNA-encoded proteins have increased their proline content during evolution (Supplementary data SD1). This observation may seem counterintuitive, as a recent evolutionary analysis of polyproline motifs across *Escherichia coli* strains indicated a selection pressure against translational stalling, especially in proteins with high translational efficiency (80). Despite the overall trend of polyproline motif loss to prevent stalling, prolines are enriched in specific protein regions, notably downstream of transmembrane helices (80). This suggests that ribosome pausing at polyproline motifs may facilitate co-translational folding and membrane insertion, a process particularly relevant for mitoribosome-synthesized proteins. Notably, we have recently reported that during the synthesis of the 12-transmembrane domain (TM) COX1 protein in mitoribosomes, translational pausing in *COX1* during the synthesis of some TMs could be regulated by the secondary structure of the *COX1* mRNA (19). The combined presence of polyprolines in the protein immediately after all TMs and hairpins in mRNA regions coding for specific chaperone-binding TMs suggest several layers of translational control to afford time for the accurate and efficient co-translational membrane insertion, which are most evident for COX1.

### Mitoribosome pausing at proline motifs is context-specific

Ribosome stalling at PP and PPP motifs is influenced by the context of the nascent polypeptide chain, particularly the amino acids directly flanking the proline residues (81–87). Studies conducted *in vitro* and in bacterial systems have identified a hierarchy of stalling PP-containing triplets. These range from strong stallers, like PPW, PPP, DPP and PPN, to weak stallers, such as CPP, PPR, and PPH, all of which are substrates for bacterial EF-P and have been assigned motif pausing scores (81–84).

There are 15 PP-containing triplets in the mtDNA-encoded proteome, only 5 of which induce stalling and are TACO1 substrates (Supplementary Table S4). Mitoribosome stalling induced by (X)PP or PP(X) followed the same rules as in bacterial ribosomes. As such, only those with medium (PPS or PPA) or high pausing scores induced stalling, but not those with weak pausing scores (PPL, PPH or TPP) (Supplementary Table S4). An exception to the rule was the PPQS* motif in ATP8, which has a medium pausing score but did not induce observable pausing (Supplementary Table S4), probably because it is one amino acid apart from the STOP codon. Regarding PPP triplets, reporter assays in *E. coli* revealed that the presence of amino acids such as C and T preceding the stall site virtually suppressed stalling at PPP motifs (24). Differently, although the occurrence of a single PPP motif in human mtDNA-encoded proteome prevents applying statistics, the motif in COX1 occurs as (G)CPPP (Supplementary Table S1) and induces the most consequential translational pause, probably due to the contribution of the preceding G.

We have also analyzed the mitoribosome stalling effects of non-consecutive P-containing motifs and TACO1-mediated alleviation. Of 13 combinations, only PSPWPL in COX3 and LPYNPN in ND2 induced pausing, which was alleviated by TACO1 (Supplementary Table S5). Motifs such as PPW and PPN have strong pausing scores (81–84), which could explain pauses in PWP and NPN motifs. However, pausing was not induced by motifs PWEP in ATP8 or PNP in ND1 (Supplementary Table S5), which illustrates the complexity of how the amino acids flanking the proline residues influence stalling. In the case of ATP8, a strong pause occurs immediately after the PWEP motif that follows as KWTKIC. However, this sequence, located at the overlapping region of ATP8 and ATP6 ORFs in their bicistronic transcript, is not a TACO1 substrate (Supplementary Figure S3C), and the stall has been attributed to the presence of a hairpin (19).

Adding further complexity, the repertoire of TACO1-target motifs is extended to instances that are not proline-rich (Supplementary Table S6). This has been reported for EF-P (87) and particularly for eIF5A, which functions more globally, not only in translation elongation but also in termination (88,89). In COX2, a TACO1-dependent pause occurred at a DYGGL motif, seven amino acids upstream of a PP sequence, and at a YGLDY motif in ND4L without nearby prolines (Supplementary Table S6). It also occurred at a QTTN sequence of an LPLPWALQTTN motif in ND3, at FASF and PAA motifs in ATP6, and at a VVV motif in ND6 (Supplementary Table S6). Overall, these data indicate that TACO1 functions as a general translation elongation factor acting upon proline-rich sequences and, more broadly, in a handful of non-proline motifs.

### Destabilization of the mitoribosome peptidyl transfer center by mutations in bL27m reveals the effect of TACO1 on most mitochondrial polypeptides

**Figure 3.**
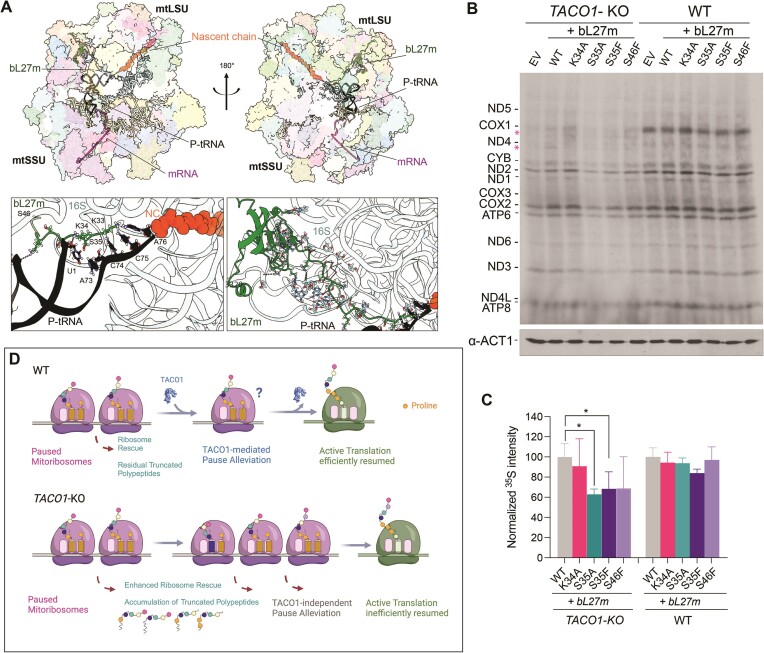
TACO1 and bL27m cooperate to stabilize the PTC during mitochondrial translation. (**A**) Cryo-EM structures of the translating mitochondrial ribosome (6ZM5) (114). P-site tRNA is depicted in black; bL27m is depicted in green; mRNA in purple; nascent chain (NC) in orange; 16S rRNA in light blue. Images were prepared in UCSF ChimeraX (115). (**B**) Metabolic labeling of newly synthesized mitochondrial translation products with ^35^S-methionine in WT and *TACO1*-KO cells carrying the empty vector (EV) or overexpressing either WT or bL27m variants carrying mutations (K34A, S35A, S35F or S46F) in its N-terminus. Immunoblot analysis against β-ACTIN is provided as a loading control. (**C**) Densitometric quantification of the newly synthesized mtDNA-encoded proteins in the genotypes reported in panel (B). Data are plotted as mean ± SD (*n* = 3 biological replicates, one-way ANOVA with Dunnet's multiple comparisons, *P ≤ 0.05). (**D**) Low-resolution model of the TACO1 function.

Studies in yeast have revealed a positive genetic interaction between *DPC29/TACO1* and the N-terminus of the mitoribosomal protein bL27m (53). While individual deletion of *DPC29* or specific mutations in bL27m does not manifest overt phenotypes, their combination results in a significant defect in mitochondrial translation. The N-terminal segment of human bL27m is rich in serine (Ser) and lysine (Lys) residues, which typically interact with RNA (Figure 3A). This domain traverses multiple helices of the 16S and extends to the mitoribosome PTC, where it directly contacts the CCA end of the P-site tRNA (Figure 3A), providing stability (Figure 3A) crucial for stabilizing the peptidyl-transferase reaction in both bacterial (90,91) and mitochondrial ribosomes (92,93). Using bacterial L27 N-terminus stabilization as a diagnostic marker of A-site tRNA accommodation (90,94), it has been shown that polyproline-induced translational stalling involves destabilization of the peptidyl-tRNA and prevents accommodation of the aa-tRNA at the A site, alterations that are resolved by EF-P (24).

To investigate whether bL27m and TACO1 collaborate to stabilize the PTC during human mitochondrial translation, we overexpressed variants of bL27 carrying mutations in N-terminal Ser/Lys residues in both WT and *TACO1*-KO backgrounds. In the WT context, mitochondrial protein synthesis rates were not affected by the bL27 mutations (Figure 3B, C). On the contrary, in the *TACO1*-KO cell lines, expression of bL27 variants carrying mutations S35A, S35F or S46F exacerbated the COX1 synthesis defect observed in the absence of TACO1 and revealed a globally attenuated translation rate (Figure 3B, C). These findings indicate that the N-terminus of bL27m and TACO1 synergistically contribute to stabilizing the PTC during translation in human mitochondria.

## Discussion

Ribosomes are highly sophisticated and efficient molecular machineries capable of synthesizing polypeptide chains thanks to their peptidyl-transferase enzymatic activity. However, ribosomes become stalled at polyproline stretches because of slow peptide bond formation between the peptidyl-Pro-tRNA in the P-site and the succeeding Pro-tRNA in the A-site (1,24). The all-trans conformation of the peptide bond in the polyproline chain clashes with the conformation of the ribosomal tunnel, destabilizing the P-site tRNA and the PTC (24). To counteract this, translation systems have evolved ancillary factors to stabilize the P-site tRNA and the PTC during polyproline synthesis. Bacterial ribosomes primarily rely on elongation factor P (EF-P), while eukaryotic systems utilize eIF5A. Despite the bacterial origin of the mitochondrial translation machinery, a homolog of EF-P is absent in mitochondria (65). Given the presence of polyproline stretches in mitochondrial DNA-encoded proteins, a factor assisting mitoribosomes in resolving polyproline-mediated stalling seems necessary.

Our research identifies the mitochondrial disease-related protein TACO1 as the translational factor that alleviates the prolonged stalling at polyproline stretches and other protein motifs in mitochondria. Moreover, we show that the pausing events occur in physiological conditions, and TACO1 is needed to assist the ribosome in efficiently resuming translation. Thus, our study provides the explanation for why the absence of TACO1 prominently affects *COX1* translation in mammals, where it is the polyproline-richest protein and contains the single tri-proline stretch in the mtDNA-encoded proteome (44,45). It also explains why no overt phenotype is observed in an *S. cerevisiae* strain deleted for the *TACO1* homolog gene *DPC29*, whose mtDNA-encoded proteome is poorer in prolines (53). Our study, therefore, sheds light on the molecular mechanism underlying TACO1-related mitochondrial encephalopathy associated with cytochrome *c* oxidase deficiency (44).

In human mitochondria, translational pausing at polyproline stretches and beyond is context-specific, following rules similar to those described for bacterial and eukaryotic ribosomes (81–89,95). Furthermore, of the 13 stalling events alleviated by TACO1 in mitochondria, only three in COX1 and two in COX3 significantly affect protein output. Studies in bacteria have highlighted the importance of initiation rates in determining the effects of pausing, with strongly expressed proteins showing the greatest dependence on EF-P (96). A subsequent study identified three factors that determine how pausing affects protein output: the strength of the pause, its location (5′ polarity), and the translational efficiency of the gene (81). In our study, we found that *COX1* has the strongest pause (PPP motif), which, although located near the 3′ of the mRNA, induces a detectable phenotype and accumulation of prematurely released polypeptides. The two other pauses in *COX1* are of minor or moderate strength (Supplementary Table S4) and, despite being in the first third of the gene, likely do not significantly contribute to the overall COX1 protein output. In mitochondria from HEK293T cells, *COX1* and *COX3* mRNAs, along with *ND6*, have the highest translation initiation rates (78), which could explain the effect of a pause at a motif near the 5′ end *COX3* mRNA in slightly attenuating COX3 protein output. Although the relatively low number of pausing events prevents robust statistical analyses, some trends can be inferred from the data. For example, pauses that do not manifest a phenotype may be due to their medium or weak strength, particularly if they occur in mRNAs with low translation initiation rates, such as *ATP8* or *ND2* (78).

Our work clarifies that TACO1 does not function as a translational activator in the manner of the recognized factors found in yeast. Unlike yeast mitochondrial mRNAs, which have large 5′-end untranslated sequences (UTRs) where activators bind, mt-mRNAs in higher eukaryotes have minimal or no 5′-UTRs (97). Therefore, we propose renaming TACO1 as ‘Translational Accelerator’ rather than ‘Translational Activator’. Further investigations are needed to uncover the precise mechanism by which TACO1 stimulates mitoribosomal peptidyltransferase activity. However, the collaboration between TACO1 and the conserved N-terminal domain of bL27m to stabilize the PTC during mitochondrial protein synthesis, akin to what has been shown in yeast (53), along with the identification of a small fraction of TACO1 specifically interacting with the mtLSU, monosomes and polysomes, could suggest that TACO1 may function similarly to EF-P and eIF5A.

In bacteria, ribosomes stalled on polyproline stretches are recognized by EF-P, which binds within the E-site region and stabilizes the peptidyl-tRNA. EF-P binding is facilitated through contacts with the LSU L1 stalk (28) and the P-site tRNA (98) as well as E-site codon (24). The interaction of the modified tip of domain 1 from EF-P with the CCA end of P-site tRNAPro stabilizes both the P-site tRNA and the nascent chain by forcing the prolines to adopt an alternative conformation that passes into the ribosomal exit tunnel (24). The mechanism of action of eIF5A is similar to that of EF-P. It functions by binding to the E-site and stabilizing the PTC, although it lacks domain 3 found in EF-P, preventing some interactions with SSU elements (23,99). Beyond EF-P and eIF5A, the ATP-binding cassette (ABC) family F (ABC-F) proteins, present in both bacteria and eukaryotes, also accelerate the translation of difficult-to-synthesize amino acid motifs (100–102). Some ABC-F proteins resolve polyproline-dependent stalling (101) and can complement the role of EF-P (103). Although their precise mechanism differs from EF-P, ABC-F proteins also interact with the E-site of ribosomes, where they make contacts with the P-site tRNA, stabilizing the PTC and facilitating peptide bond formation (104,105).

Notably, TACO1 exhibits an overall L-shape (45) reminiscent of a tRNA, akin to EF-P (106), but with a slightly more hooked configuration (Supplementary Figure S5). The structure of TACO1 differs from EF-P (24,28,106), eIF5A (23,107,108) or ABC-F proteins, yet shares a similar electrostatic charge distribution (Supplementary Figure S5), suggesting it may function through mechanisms analogous to EF-P, eIF5A or ABC-F by binding to an empty E-site. A proposed model is presented in Figure 3D. However, our Bio-ID studies suggest that TACO1 could bind to the mitoribosome near the L7/L12 stalk, implying a different mechanism of action, possibly involving the A-site. Future structural investigations are warranted to decipher the details of mitoribosome stalling by polyproline stretches and other motifs, and their rescue by TACO1.

Given the presence of EF-P in bacteria, the co-existence of TACO1 homologs, specifically the YebC-family of proteins, is intriguing. Although the molecular function of these proteins remains elusive, several studies have underscored their significance in bacterial virulence and quorum sensing (109–111). For instance, YebC can rescue the cold-sensitive phenotype induced by the loss of *BipA*, a member of the ribosome-binding translational GTPase superfamily, including canonical elongation factors like EF-Tu and EF-G, functioning as a global stress-responsive regulator of protein synthesis at low temperatures (112). Furthermore, *BipA* deletions are also rescued by mutations in *rluC*, which encodes a pseudouridine synthase targeting specific sites of the 23S rRNA surrounding the PTC (113). These findings suggest the YebC-family proteins might function as EF-P-like factors in bacteria, a role they could perform under specific environmental conditions or to cope with specific amino acid sequence contexts flanking polyproline stretches or other stalling motifs in nascent peptide sequences within the exit tunnel.

## Supplementary Material

gkae645_Supplemental_Files

## Data Availability

All Source Data is included in the manuscript or will be provided upon request. Ribosome profiling sequencing data are available at EMBL-EBI BioStudies with link: https://www.ebi.ac.uk/biostudies/arrayexpress/studies/E-MTAB-14071. Mass spectrometry data have been deposited to PRoteomics IDEntifications Database (PRIDE, https://www.ebi.ac.uk/pride/) with project name “Human mitochondrial TACO1-FLAG SILAC AP-MS”, accession number PXD051090, and Project DOI: 10.6019/PXD051090.
