# Supplementary material for: The human mitochondrial translation factor TACO1 alleviates mitoribosome stalling at polyproline stretches
Source: Nucleic Acids Res. 2024 Jul 22;52(16):9710–26. doi: 10.1093/nar/gkae645 (PMC11381339; doi:10.1093/nar/gkae645)
Supplement: gkae645_Supplemental_Files [file gkae645_supplemental_files.zip › Supplementary table S2.pdf]

Supplementary table S2 - IP

Protein ID (new nomenclature for mitoribosome proteins)	Log2 Fold Change by Category (TACO1_Flag / Reference)	T-Test (p-value) Benjamini-Hochberg (p < 0.00797)	Significance (-log10)
TACO1	5.83	0.0021	2.677780705
MGARP	1.17	0.12	0.920818754
RDH13	1.09	0.6	0.22184875
SFXN2	0.89	0.029	1.537602002
ATP5IF1	0.87	--	#VALUE!
MDH2	0.86	0.38	0.420216403
MCUB	0.78	--	#VALUE!
DGUOK	0.7	--	#VALUE!
CHCHD6	0.69	0.00011	3.958607315
SFXN4	0.67	0.00029	3.537602002
POLQ	0.67	--	#VALUE!
GPD2	0.65	0.00011	3.958607315
PRDX2	0.65	0.83	0.080921908
RDH14	0.63	--	#VALUE!
CCDC127	0.61	--	#VALUE!
SLC25A5	0.6	0.01	2
VDAC3	0.6	0.35	0.455931956
MTX1	0.55	--	#VALUE!
MTCH1	0.54	0.011	1.958607315
ROMO1	0.52	0.71	0.148741651
MGST3	0.49	0.15	0.823908741
CCDC51	0.49	--	#VALUE!
NDUFB4	0.48	0.021	1.677780705
MRPL30 (uL30m)	0.48	0.15	0.823908741
NDUFA1	0.48	--	#VALUE!
NSUN4	0.47	0.29	0.537602002
FOXRED1	0.45	0.055	1.259637311
MRPL10 (uL10m)	0.44	0.14	0.853871964
SLC25A19	0.43	0.032	1.494850022
ATP5F1B	0.42	0.0073	2.13667714
RHOT1	0.42	0.013	1.886056648
DMAC2	0.42	0.056	1.251811973
BNIP3	0.42	--	#VALUE!
MICOS13	0.4	0.17	0.769551079
SLC30A9	0.4	0.047	1.327902142

MRPL32 (bL32m)	0.4	--	#VALUE!
SLC25A13	0.39	0.0063	2.200659451
ABCB7	0.39	0.017	1.769551079
FAM162A	0.39	0.0039	2.408935393
ACSM2A	0.39	--	#VALUE!
HADHB	0.38	0.02	1.698970004
SLC25A6	0.37	0.028	1.552841969
AIFM1	0.37	0.00043	3.366531544
ATP5PO	0.37	0.0024	2.619788758
PNKD	0.37	0.17	0.769551079
RMDN3	0.36	0.0029	2.537602002
MRPL33 (bL33m)	0.36	0.092	1.036212173
NDUFB1	0.36	0.0037	2.431798276
OCIAD1	0.35	0.014	1.853871964
NDUFC2	0.35	0.0093	2.031517051
MRPL37 (mL37)	0.34	0.00097	3.013228266
MT-ATP6	0.34	0.27	0.568636236
SMIM8	0.34	0.39	0.408935393
HADHA	0.33	0.023	1.638272164
SLC25A14	0.33	0.04	1.397940009
CHCHD3	0.32	0.014	1.853871964
MT-ND2	0.32	0.0034	2.468521083
SLC25A4	0.31	0.11	0.958607315
MRPL21 (bL21m)	0.31	0.0018	2.744727495
MRPL51 (mL51)	0.31	--	#VALUE!
MRPL43 (mL43)	0.3	0.01	2
IMMT	0.29	0.009	2.045757491
ATP5MG	0.29	0.023	1.638272164
OPA1	0.29	0.03	1.522878745
MRPS18A (mL66)	0.29	0.00062	3.207608311
MCU	0.29	0.11	0.958607315
NME4	0.29	--	#VALUE!
LYRM4	0.29	0.014	1.853871964
ECSIT	0.28	--	#VALUE!
YME1L1	0.27	0.0038	2.420216403
MRPL1 (uL1m)	0.27	0.19	0.721246399
MTX2	0.27	0.0089	2.050609993
NDUFS5	0.27	< 0.0001	#VALUE!
PARL	0.27	0.51	0.292429824
COX7A2	0.27	0.56	0.251811973

MAIP1	0.27	0.48	0.318758763
NT5DC2	0.27	--	#VALUE!
TIMM17B	0.26	0.24	0.619788758
NDUFB11	0.26	0.0084	2.075720714
STOM	0.26	0.41	0.387216143
ATP5F1A	0.25	0.0046	2.337242168
LETM1	0.25	0.0074	2.13076828
EXOG	0.25	0.92	0.036212173
ATP5PB	0.24	0.0092	2.036212173
MRPL11 (uL11m)	0.24	0.04	1.397940009
MRPL13 (uL13m)	0.24	0.034	1.468521083
MRPL49 (mL49)	0.24	0.074	1.13076828
NDUFA10	0.24	0.012	1.920818754
MRPL55 (bL31m)	0.24	0.38	0.420216403
MRPL34 (bL34m)	0.24	0.014	1.853871964
AGK	0.23	0.038	1.420216403
DNAJC11	0.23	0.029	1.537602002
SLC25A12	0.23	0.0014	2.853871964
MRPL28 (bL28m)	0.23	< 0.0001	#VALUE!
SLC25A22	0.23	0.0052	2.283996656
SDHA	0.23	0.067	1.173925197
MRPL27 (bL27m)	0.23	0.053	1.27572413
ALDH3A2	0.23	0.37	0.431798276
SCO1	0.23	0.21	0.677780705
FAM210B	0.23	0.74	0.13076828
MRPL42 (mL42)	0.23	0.64	0.193820026
SAMM50	0.22	0.12	0.920818754
NDUFA13	0.22	0.0073	2.13667714
MRPL48 (mL48)	0.22	0.17	0.769551079
NDUFA2	0.22	0.16	0.795880017
NDUFV1	0.21	0.48	0.318758763
NDUFB5	0.21	0.013	1.886056648
NSUN2	0.21	--	#VALUE!
OXA1L	0.2	0.0004	3.397940009
MRPL23 (uL23m)	0.2	0.035	1.455931956
MTPAP	0.2	0.2	0.698970004
COA1	0.2	0.00011	3.958607315
MRPL57 (mL63)	0.2	0.0037	2.431798276
MRPL47 (uL29m)	0.19	0.14	0.853871964
ACAD9	0.19	0.23	0.638272164

MRPL58 (mL62)	0.19	0.041	1.387216143
MT-ATP8	0.19	0.011	1.958607315
NDUFB7	0.19	0.54	0.26760624
ATP5F1C	0.18	0.032	1.494850022
MRPL45 (mL45)	0.18	0.034	1.468521083
MT-ND4	0.18	0.059	1.229147988
MRPL14 (uL14m)	0.18	0.011	1.958607315
ATP5ME	0.18	0.0034	2.468521083
SLC25A36	0.18	0.67	0.173925197
TMEM126A	0.18	0.24	0.619788758
ABCB10	0.18	0.79	0.102372909
MMAB	0.18	--	#VALUE!
COX15	0.17	0.0025	2.602059991
ATP5PD	0.17	0.1	1
NDUFA9	0.17	0.19	0.721246399
GHITM	0.17	< 0.0001	#VALUE!
MRPL22 (uL22m)	0.17	0.11	0.958607315
MRPL4 (uL4m)	0.17	0.23	0.638272164
GADD45GIP1	0.17	0.15	0.823908741
MRPL2 (uL2m)	0.17	0.093	1.031517051
ATP5F1E	0.17	0.11	0.958607315
MRPS30 (mL65)	0.17	0.29	0.537602002
MICU2	0.17	--	#VALUE!
MRPL39 (mL39)	0.16	0.56	0.251811973
MRPL12 (bL12m)	0.16	0.06	1.22184875
NDUFS8	0.16	0.25	0.602059991
MRPL9 (bL9m)	0.16	0.02	1.698970004
NDUFAF3	0.16	0.021	1.677780705
NDUFS1	0.15	0.55	0.259637311
MRPL16 (uL16m)	0.15	0.048	1.318758763
NDUFA8	0.15	0.0015	2.823908741
PRKACA	0.15	0.8	0.096910013
NDUFV3	0.15	0.77	0.113509275
CLPB	0.14	0.32	0.494850022
MRPL19 (bL19m)	0.14	0.038	1.420216403
MRPL24 (uL24m)	0.14	0.15	0.823908741
TIMM50	0.14	0.44	0.356547324
ATAD3A	0.13	0.02	1.698970004
MRPL18 (uL18m)	0.13	0.46	0.337242168
DNAJC19	0.13	0.17	0.769551079

ECHS1	0.13	0.57	0.244125144
ATP5F1D	0.13	0.028	1.552841969
PHB2	0.12	0.082	1.086186148
PAM16	0.12	0.0038	2.420216403
MRPL54 (mL54)	0.12	0.67	0.173925197
MRPL53 (mL53)	0.12	0.28	0.552841969
DNAJC30	0.12	0.12	0.920818754
MRPL17 (bL17m)	0.11	0.17	0.769551079
MRPL44 (mL44)	0.11	0.24	0.619788758
MRPL46 (mL46)	0.11	0.32	0.494850022
UQCRC2	0.11	0.83	0.080921908
MTCH2	0.11	0.22	0.657577319
NDUFS2	0.1	0.59	0.229147988
TOMM20	0.1	0.031	1.508638306
ATP5MF	0.1	0.5	0.301029996
PGAM5	0.09	0.043	1.366531544
NDUFS7	0.09	0.34	0.468521083
MRPL40 (mL40)	0.09	0.55	0.259637311
NDUFA5	0.09	0.59	0.229147988
MRPL41 (mL41)	0.09	0.58	0.236572006
ATP5PF	0.09	0.72	0.142667504
SLC25A30	0.09	0.16	0.795880017
NDUFB8	0.09	0.27	0.568636236
AFG3L2	0.08	0.65	0.187086643
NDUFS3	0.08	0.45	0.346787486
MRPL3 (uL3m)	0.08	0.68	0.167491087
NDUFS4	0.08	0.64	0.193820026
ATAD1	0.08	0.79	0.102372909
DHRS7B	0.08	--	#VALUE!
MT-ND1	0.08	0.65	0.187086643
EXD2	0.08	0.89	0.050609993
NDUFV2	0.07	0.44	0.356547324
MPC2	0.07	0.78	0.107905397
MRPL38 (mL38)	0.06	0.72	0.142667504
LETMD1	0.06	0.47	0.327902142
CHCHD4	0.06	0.17	0.769551079
NDUFA4	0.05	0.18	0.744727495
HCCS	0.05	0.4	0.397940009
MRPL20 (bL20m)	0.05	0.52	0.283996656
MT-CO2	0.05	0.58	0.236572006

MRPL15	0.04	0.64	0.193820026
NDUFA12	0.04	0.92	0.036212173
TIMM29	0.04	0.91	0.040958608
MPV17L2	0.04	0.88	0.055517328
NDUFAF4	0.03	0.66	0.180456064
SLC25A15	0.03	0.53	0.27572413
HIGD2A	0.02	0.89	0.050609993
PXMP2	0.02	--	#VALUE!
TFAM	0.02	--	#VALUE!
MRPL50 (mL50)	0.01	0.87	0.060480747
SFXN1	0	0.67	0.173925197
RHOT2	0	--	#VALUE!
SDHB	0	0.81	0.091514981
CAT	0	--	#VALUE!
SLC25A21	0	--	#VALUE!
DDX28	0	--	#VALUE!
RPUSD4	0	--	#VALUE!
TOMM7	0	--	#VALUE!
NADK2	0	--	#VALUE!
MRPS18C (bS18m)	0	--	#VALUE!
COX11	0	--	#VALUE!
LONP1	0	--	#VALUE!
FECH	0	--	#VALUE!
PPA2	0	--	#VALUE!
GRPEL1	0	--	#VALUE!
DCAKD	0	--	#VALUE!
HDHD5	0	--	#VALUE!
PLGRKT	0	--	#VALUE!
MTERF3	0	--	#VALUE!
KARS1	0	--	#VALUE!
PITRM1	0	--	#VALUE!
MAVS	0	--	#VALUE!
PNPT1	0	--	#VALUE!
DIABLO	0	--	#VALUE!
PDK1	0	--	#VALUE!
NDUFA3	0	--	#VALUE!
AHCYL1	0	--	#VALUE!
PDK3	0	--	#VALUE!
DUT	0	--	#VALUE!
TRMT61B	0	--	#VALUE!

GPX1	0	--	#VALUE!
PET100	0	--	#VALUE!
IBA57	0	--	#VALUE!
CISD1	0	--	#VALUE!
GLRX5	0	--	#VALUE!
SPTLC2	0	--	#VALUE!
NME3	0	--	#VALUE!
UQCRH	0	--	#VALUE!
TMEM126B	0	--	#VALUE!
VDAC2	-0.01	0.89	0.050609993
PISD	-0.01	0.83	0.080921908
NDUFB6	-0.01	0.89	0.050609993
FAM210A	-0.02	0.98	0.008773924
NDUFB10	-0.03	0.14	0.853871964
TAMM41	-0.03	0.3	0.522878745
TIMM21	-0.04	0.83	0.080921908
NDUFB3	-0.04	0.85	0.070581074
NDUFA11	-0.04	0.87	0.060480747
SPG7	-0.04	0.29	0.537602002
MTX3	-0.04	0.77	0.113509275
APOOL	-0.05	0.91	0.040958608
UQCRFS1	-0.05	0.56	0.251811973
NDUFB9	-0.05	0.82	0.086186148
TIMM10	-0.05	--	#VALUE!
ATAD3B	-0.06	0.82	0.086186148
MRM3	-0.06	0.85	0.070581074
LACTB	-0.06	0.99	0.004364805
COX16	-0.07	--	#VALUE!
SLC25A3	-0.08	0.55	0.259637311
ARF5	-0.08	0.21	0.677780705
COX5B	-0.08	0.76	0.119186408
SLC25A11	-0.09	0.033	1.48148606
VDAC1	-0.1	0.19	0.721246399
COX4I1	-0.11	0.11	0.958607315
HIGD1A	-0.12	0.31	0.508638306
COA3	-0.12	0.72	0.142667504
COX18	-0.12	0.28	0.552841969
POLDIP2	-0.12	0.61	0.214670165
COX5A	-0.12	--	#VALUE!
MALSU1	-0.13	0.35	0.455931956

TOMM40	-0.13	0.92	0.036212173
TIMM23	-0.14	0.22	0.657577319
OAT	-0.15	0.42	0.37675071
MRS2	-0.16	0.0055	2.259637311
NDUFA6	-0.17	0.32	0.494850022
MYO19	-0.18	0.73	0.13667714
UQCRC1	-0.18	0.67	0.173925197
CLPX	-0.18	--	#VALUE!
CYCS	-0.19	0.066	1.180456064
SARS2	-0.19	0.85	0.070581074
NDUFA7	-0.19	0.91	0.040958608
MT-CO3	-0.19	--	#VALUE!
POLRMT	-0.21	0.012	1.920818754
PDHX	-0.21	0.28	0.552841969
HSD17B10	-0.22	0.015	1.823908741
COX7A2L	-0.22	0.17	0.769551079
STOML2	-0.23	0.35	0.455931956
TIMM44	-0.23	0.58	0.236572006
MTHFD2	-0.23	0.74	0.13076828
TMEM177	-0.24	0.0041	2.387216143
SNAP29	-0.24	--	#VALUE!
HSPD1	-0.25	0.0048	2.318758763
TUFM	-0.25	0.00087	3.060480747
SSBP1	-0.25	0.32	0.494850022
TIMMDC1	-0.26	0.014	1.853871964
PDF	-0.26	0.14	0.853871964
MRPS6 (bS6m)	-0.27	0.049	1.30980392
SLIRP	-0.28	0.13	0.886056648
PMPCA	-0.29	0.086	1.065501549
ACOT9	-0.29	0.035	1.455931956
GTPBP10	-0.29	0.26	0.585026652
LRPPRC	-0.31	0.00023	3.638272164
TRAP1	-0.31	0.023	1.638272164
DHX30	-0.32	0.00059	3.229147988
DNAJA3	-0.32	< 0.0001	#VALUE!
ABCD3	-0.32	0.28	0.552841969
GRSF1	-0.33	0.0054	2.26760624
CYC1	-0.33	0.017	1.769551079
ME2	-0.33	--	#VALUE!
PRDX3	-0.34	0.01	2

UQCRQ	-0.34	0.14	0.853871964
C1QBP	-0.35	0.00057	3.244125144
MRPS22 (mS22)	-0.35	0.0045	2.346787486
YARS2	-0.35	--	#VALUE!
HSPA9	-0.37	< 0.0001	#VALUE!
MRPS34 (mS34)	-0.37	0.019	1.721246399
MRPS31 (mS31)	-0.4	< 0.0001	#VALUE!
COX7B	-0.4	0.52	0.283996656
UQCR10	-0.41	0.045	1.346787486
PPIF	-0.41	--	#VALUE!
MRPS33 (mS33)	-0.42	0.038	1.420216403
TMEM70	-0.43	0.00054	3.26760624
MRPS25 (mS25)	-0.43	< 0.0001	#VALUE!
MRPS9 (uS9m)	-0.45	< 0.0001	#VALUE!
MT-ND5	-0.45	0.4	0.397940009
MRPS2 (uS2m)	-0.46	< 0.0001	#VALUE!
PTCD1	-0.46	0.2	0.698970004
TRUB2	-0.46	--	#VALUE!
MRPS7	-0.47	< 0.0001	#VALUE!
MRPS18B (mS40)	-0.47	0.00013	3.886056648
SND1	-0.47	--	#VALUE!
ACAT1	-0.48	0.00033	3.48148606
MRPS26 (mS26)	-0.48	< 0.0001	#VALUE!
DLAT	-0.48	0.002	2.698970004
MRPS17 (uS17m)	-0.49	0.0017	2.769551079
AKAP1	-0.5	0.0016	2.795880017
NDUFS6	-0.5	0.21	0.677780705
PTCD3	-0.51	0.003	2.522878745
MRPS24 (uS3m)	-0.51	< 0.0001	#VALUE!
MRPS27 (mS27)	-0.52	0.011	1.958607315
MRPS23 (mS23)	-0.52	0.0023	2.638272164
MRPS5 (uS5m)	-0.52	0.00039	3.408935393
MRPS10 (uS10m)	-0.52	0.00046	3.337242168
MRPS21 (bS21m)	-0.53	0.038	1.420216403
MRPS16 (bS16m)	-0.55	0.004	2.397940009
MRPS12 (uS12m)	-0.55	< 0.0001	#VALUE!
IDH3B	-0.56	0.24	0.619788758
TIMM9	-0.56	--	#VALUE!
MRPS28 (bS1m)	-0.57	0.0062	2.207608311
DAP3 (mS39)	-0.58	0.011	1.958607315

HSPE1	-0.58	0.00035	3.455931956
MRPS14 (uS14m)	-0.6	< 0.0001	#VALUE!
OMA1	-0.6	0.099	1.004364805
COX6C	-0.61	0.033	1.48148606
MRPS35 (mS35)	-0.62	< 0.0001	#VALUE!
SCO2	-0.62	< 0.0001	#VALUE!
GLUD1	-0.64	< 0.0001	#VALUE!
PDHB	-0.65	--	#VALUE!
TRMT10C	-0.66	0.0014	2.853871964
SLC25A10	-0.66	0.12	0.920818754
MCCC2	-0.67	--	#VALUE!
RPUSD3	-0.68	0.054	1.26760624
MRPS15 (uS15m)	-0.7	0.0015	2.823908741
TFB1M	-0.71	0.00076	3.119186408
MRPS11 (uS11m)	-0.74	0.011	1.958607315
SLC25A1	-0.83	< 0.0001	#VALUE!
SLC25A33	-0.83	0.0051	2.292429824
PDHA1	-0.86	0.003	2.522878745
CKMT2	-0.86	--	#VALUE!
PYCR2	-0.89	--	#VALUE!
ERAL1	-0.95	0.012	1.920818754
UQCRB	-0.98	0.0011	2.958607315
ALDH18A1	-0.99	< 0.0001	#VALUE!
COQ8A	-1.01	--	#VALUE!
MICU1	-1.07	0.13	0.886056648
MTHFD1L	-1.09	< 0.0001	#VALUE!
COX6B1	-1.15	--	#VALUE!
MCAT	-1.18	0.027	1.568636236
CHCHD1	-1.31	--	#VALUE!
NOA1	-1.32	--	#VALUE!
NNT	-1.47	--	#VALUE!
AURKAIP1	-1.73	--	#VALUE!
DLST	-2.01	--	#VALUE!
