# Supplementary material for: The human mitochondrial translation factor TACO1 alleviates mitoribosome stalling at polyproline stretches
Source: Nucleic Acids Res. 2024 Jul 22;52(16):9710–26. doi: 10.1093/nar/gkae645 (PMC11381339; doi:10.1093/nar/gkae645)
Supplement: gkae645_Supplemental_Files [file gkae645_supplemental_files.zip › Supplementary table S3.pdf]

Supplementary table S3 – BioID

Bait	PreyGene (name name for mitoribosome proteins)	AvgSpec	Specificity
TACO1	MRPS28 (bS1m)	349.04	4.56
TACO1	MRPS18A (mL66)	18.21	4.36
TACO1	MRPS2 (uS2m)	156.76	4.32
TACO1	OXA1L	6.64	4.14
TACO1	DBT	147.74	4.02
TACO1	LYRM4	28.02	3.99
TACO1	MRPL12 (bL12m)	53.7	3.83
TACO1	MRPS6 (bS6m)	160.75	3.79
TACO1	MRPL42 (mL42)	34.12	3.71
TACO1	NDUFS6	90.48	3.7
TACO1	MRPL10 (uL10m)	20.52	3.67
TACO1	MRPS10 (uS10m)	74.85	3.64
TACO1	MRPS33 (mS33)	76.98	3.62
TACO1	OGDH	59.82	3.6
TACO1	MRPS18B (mS40)	100.81	3.57
TACO1	NT5DC2	17.11	3.55
TACO1	DAP3 (mS39)	133.27	3.54
TACO1	MRPS18C (bS18m)	28.73	3.54
TACO1	PDHA1	25.94	3.54
TACO1	MRPS14 (uS14m)	65.74	3.46
TACO1	NME4	59.73	3.42
TACO1	PYCR1	64.35	3.4
TACO1	PYCR2	90.11	3.4
TACO1	TSFM	4.61	3.4
TACO1	MRPS36	106.46	3.39
TACO1	MRPL19 (bL19m)	20.09	3.37
TACO1	MRPL58 (mL62)	14.85	3.33
TACO1	C17orf80	23.14	3.32
TACO1	MRPS9 (uS9m)	117.82	3.32
TACO1	PDHX	7.38	3.32
TACO1	PTCD3	86.59	3.32
TACO1	THG1L	4.28	3.32
TACO1	MRPS17 (uS17m)	56.88	3.3
TACO1	MRPS7 (uS7m)	122.19	3.28
TACO1	GATC	11.25	3.26
TACO1	MRPL13 (uL13m)	38.68	3.24
TACO1	MRPS23 (mS23)	178.5	3.14

TACO1	MRPS31 (mS31)	113.62	3.14
TACO1	MRPS35 (mS35)	103.42	3.13
TACO1	PDHB	23.38	3.11
TACO1	MRPS34 (mS34)	146.17	3.1
TACO1	NDUFA7	78.98	3.09
TACO1	MRPS5 (uS5m)	83.57	3.08
TACO1	MRPL18 (uL18m)	19.83	3.07
TACO1	CS	9.28	3.04
TACO1	ETFB	23	3.04
TACO1	HINT2	20.34	3.03
TACO1	MRPL43 (mL43)	34.4	3.01
TACO1	MRPL51 (mL51)	11.95	3.01
TACO1	GADD45GIP1	19.53	3
TACO1	GATB	7.78	3
TACO1	MRPL16 (uL16m)	7.07	2.99
TACO1	MRPS16 (bS16m)	76.31	2.99
TACO1	NDUFA6	44.71	2.98
TACO1	ALAS1	7.17	2.93
TACO1	NDUFAB1	9.81	2.93
TACO1	MRPS27 (mS27)	103.48	2.9
TACO1	MRPL39 (mL39)	16.61	2.88
TACO1	MRPS22 (mS22)	135.26	2.88
TACO1	MRPL2 (uL2m)	9.2	2.87
TACO1	NDUFAF2	60.36	2.85
TACO1	DLST	52.82	2.84
TACO1	IARS2	37.19	2.84
TACO1	PAM16	22.44	2.84
TACO1	NDUFA12	89.69	2.8
TACO1	QRSL1	7.73	2.8
TACO1	MRPL3 (uL3m)	16.12	2.77
TACO1	MRPS26 (mS26)	104.11	2.77
TACO1	AK4	16.01	2.72
TACO1	NFS1	17.25	2.72
TACO1	SHMT2	128.22	2.72
TACO1	ACOT1	110.54	2.68
TACO1	ACOT2	106.87	2.66
TACO1	KIAA0391	3.09	2.66
TACO1	HSDL2	8.57	2.65
TACO1	POLDIP2	13.6	2.65
TACO1	MRPL53 (mL53)	24.86	2.64

TACO1	LETM1	36.54	2.62
TACO1	MRPL20 (bL20m)	35.87	2.62
TACO1	MRPL45 (mL45)	27.47	2.62
TACO1	MMAB	27.54	2.61
TACO1	MTERF3	8.56	2.6
TACO1	NDUFA2	51.52	2.6
TACO1	MRPL17 (bL17m)	23.31	2.59
TACO1	MRPL55 (bL31m)	12.44	2.59
TACO1	MRPS30 (mL65)	9.87	2.59
TACO1	CLPX	20.48	2.56
TACO1	GLS	25.13	2.55
TACO1	MRPL52 (mL52)	16.59	2.54
TACO1	NDUFS7	27.44	2.54
TACO1	MRPL47 (uL29m)	23.46	2.53
TACO1	MRPS24 (uS3m)	85.51	2.53
TACO1	NDUFAF1	10.92	2.53
TACO1	MRPL50 (mL50)	33.89	2.52
TACO1	ECHS1	22.86	2.51
TACO1	MRPL46 (mL46)	21.02	2.5
TACO1	RBMX	32.61	2.5
TACO1	LRPPRC	40.73	2.49
TACO1	PDK3	6.69	2.48
TACO1	LARS2	4.41	2.47
TACO1	NDUFA9	69.84	2.46
TACO1	MRPL1 (uL1m)	15.69	2.45
TACO1	TWNK	9.64	2.43
TACO1	ELAC2	10.7	2.42
TACO1	TRMT10C	36.55	2.42
TACO1	MRPS25 (mS25)	254.71	2.41
TACO1	DLAT	12.55	2.39
TACO1	MDH2	9.81	2.39
TACO1	NIPSNAP1	12.46	2.39
TACO1	ETFA	30.56	2.38
TACO1	HNRNPL	54.78	2.36
TACO1	MRPL24 (uL24m)	25.78	2.36
TACO1	MRPL41 (mL41)	36.52	2.35
TACO1	MRPL23 (uL23m)	19.93	2.34
TACO1	NDUFA5	93.25	2.34
TACO1	VWA8	9.77	2.34
TACO1	METTL15	3.11	2.33

TACO1	MRPL27 (bL27m)	22.4	2.32
TACO1	MRPL37 (mL37)	17.3	2.31
TACO1	MRPL40 (mL40)	13.62	2.29
TACO1	MRPS11 (uS11m)	17.43	2.26
TACO1	ATP5PO	16.26	2.25
TACO1	BCS1L	15.82	2.25
TACO1	MRPL49 (mL49)	19.97	2.25
TACO1	TIMM44	29.81	2.25
TACO1	MRM3	10.93	2.24
TACO1	NDUFS1	50.81	2.22
TACO1	NDUFV1	35.72	2.22
TACO1	NDUFS4	34.97	2.2
TACO1	SUCLA2	7.41	2.2
TACO1	MRPL4	23.78	2.19
TACO1	NDUFV3	67.39	2.18
TACO1	IBA57	8.12	2.15
TACO1	NDUFV2	25.6	2.13
TACO1	MRPL48	14.43	2.09
TACO1	CARS2	6.33	2.08
TACO1	GTPBP10	12.52	2.07
TACO1	MRPL21 (bL21m)	23.63	2.07
TACO1	COX5A	15.16	2.06
TACO1	STOML2	12.86	2.02
TACO1	ATP5PB	15.6	2.01
TACO1	AFG3L2	24.54	2
TACO1	MRPL44 (mL44)	23.72	1.99
TACO1	NDUFS2	77.08	1.98
TACO1	NDUFS8	28.95	1.94
TACO1	NDUFAF3	11.09	1.9
TACO1	VARS2	5.44	1.89
TACO1	NDUFS3	72.08	1.87
TACO1	PPIF	11.09	1.87
TACO1	MRPL15 (uL15m)	18.95	1.81
TACO1	RTN4IP1	5.15	1.77
TACO1	ERAL1	9.27	1.76
TACO1	MRPL38 (mL38)	8.72	1.75
TACO1	TRUB2	3.08	1.74
TACO1	MRPL28 (bL28m)	20.92	1.72
TACO1	COX4I1	11.89	1.71
TACO1	ATP5F1B	113.94	1.69

TACO1	ATPAF1	13.08	1.68
TACO1	MRPL22 (uL22m)	10.99	1.61
TACO1	SLIRP	42.11	1.61
TACO1	NDUFAF4	26.23	1.6
TACO1	SLC30A9	5.84	1.58
TACO1	GRSF1	35.19	1.56
TACO1	HSD17B10	68.76	1.54
TACO1	PDE12	2.09	1.54
TACO1	ATP5MF-PTCD1	5.2	1.49
TACO1	POLRMT	7.6	1.48
TACO1	ATP5PF	11.81	1.47
TACO1	MRPL9	7.64	1.36
TACO1	FASTKD2	3.91	1.16
TACO1	ECSIT	3.55	1.14
TACO1	DHX30	9.26	1.1
TACO1	ACAT1	3.56	1.01
TACO1	PHB	53.36	0.97
TACO1	PNPT1	2.61	0.95
TACO1	ACAD9	2.05	0.38
