# Supplementary material for: The human mitochondrial translation factor TACO1 alleviates mitoribosome stalling at polyproline stretches
Source: Nucleic Acids Res. 2024 Jul 22;52(16):9710–26. doi: 10.1093/nar/gkae645 (PMC11381339; doi:10.1093/nar/gkae645)
Supplement: gkae645_Supplemental_Files [file gkae645_supplemental_files.zip › TACO1-Supplementary-NAR-revision.pdf]

Supplementary Figures and Tables for:

The human mitochondrial translation factor TACO1 alleviates mitoribosome stalling at polyproline stretches

Michele Brischigliaro[1#], Annika Krüger[2,3#], J. Conor Moran[4], Hana Antonicka[5], Ahram Ahn[4], Eric A. Shoubridge[5], Joanna Rorbach[2,3*], and Antoni Barrientos[1,4,6*¶]

[1] Department of Neurology. University of Miami Miller School of Medicine. 1600 NW 10th Ave. Miami, FL-33136, USA.
[2] Department of Medical Biochemistry and Biophysics, Karolinska Institutet, Stockholm, Sweden.
[3] Max Planck Institute Biology of Ageing-Karolinska Institutet Laboratory, Karolinska Institutet, Stockholm, Sweden.
[4] Department of Biochemistry and Molecular Biology. University of Miami Miller School of Medicine. 1600 NW 10th Ave. Miami, FL-33136, USA.
[5] The Neuro and Department of Human Genetics, McGill University, Montreal, QC, Canada
[6] The Miami Veterans Affairs (VA) Medical System. 1201 NW 16th St, Miami, FL-33125, USA.

[*]Correspondence to:
Antoni Barrientos: abarrientos@med.miami.edu
Joanna Rorbach: joanna.rorbach@ki.se

Supplementary Figures

Supplementary Figure S1. The extra polypeptides that are newly synthesized in the absence of TACO1 are aberrant products of COX1 mRNA translation. Related to Fig. 1.

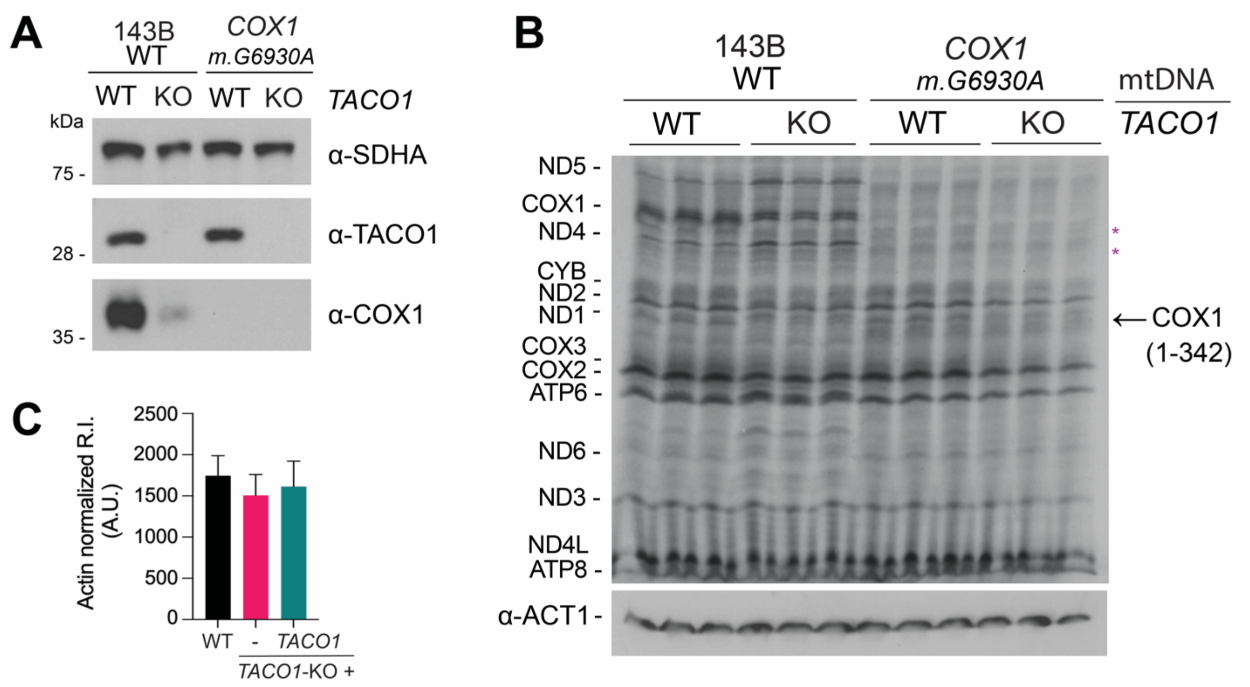

(A) Steady-state levels of TACO1, COX1, and SDHA in WT 143B cells and *COX1-m.G6930A* homoplasmic cybrids in which *TACO1* has been knocked out. **(B)** Metabolic labeling of de novo synthesized mitochondrial translation products with ^{35}S-methionine as in Figure 1C in 143B cells with the same genotypes as in (A). Asterisks indicate truncated products of COX1 translation. Immunoblot analysis against β- ACTIN is provided as a loading control. **(C)** Relative intensity of the ^{35}S-methionine signal from fully synthesized COX1 and aberrant mitochondrial translation products, estimated as in Fig. 1C). Data are plotted as mean ± SD (n = 3 biological replicates, one-way ANOVA, not significant).

Supplementary Figure S2. A minute fraction of TACO1 co-sediments with mitoribosomes and its overexpression enhances the overall rate of translation. Related to Fig. 2.

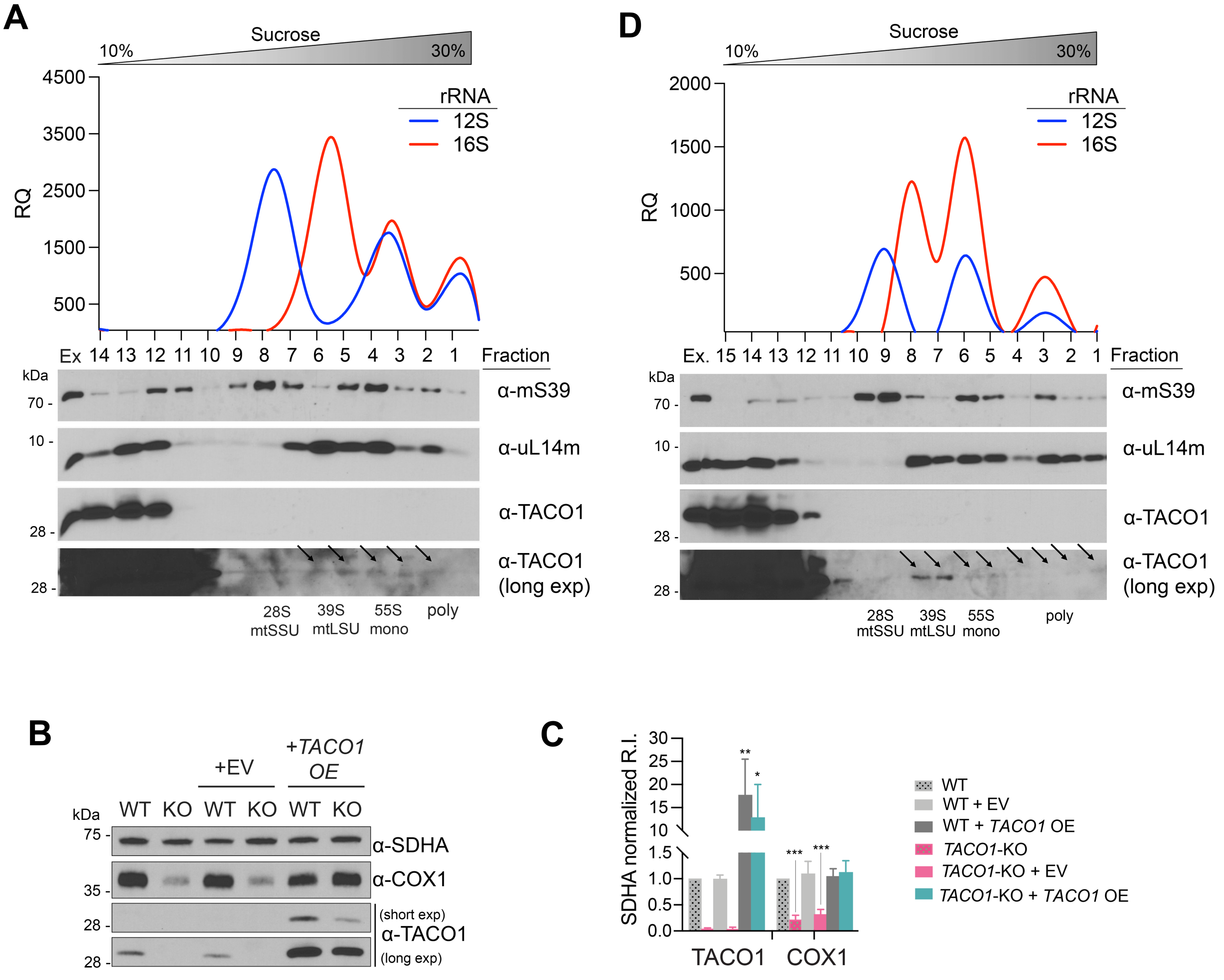

(A-D) Sucrose gradient sedimentation analyses of TACO1 and mtSSU (mS39) and mtLSU markers (uL14m) in mitochondria purified from WT **(A)** and *TACO1* OE **(D)** cells and extracted in the presence 20 mM $MgCl_2$, to preserve the integrity of monosomes and polysomes. The fractions were used to measure 12S rRNA and 16S rRNA concentrations (top) by RT-qPCR (see details in the Methods section). **(B)** Steady-state levels of TACO1, COX1, and SDHA in WT and *TACO1*-KO cells overexpressing TACO1. Either naïve or empty vector-carrying WT and *TACO1*-KO cells were included as controls. **(C)** Densitometric quantitative analysis of the steady-state levels of TACO1, COX1, and SDHA reported in (B). Data are plotted as mean ± SD (n = 3 biological replicates, one-way ANOVA with Sidak's multiple comparisons, ***$p \leq 0.001$).

Supplementary Figure S3. Analysis of mitoribosome profiling data of WT, *TACO1*-KO and *TACO1*-KO reconstituted cells. Related to Fig. 2

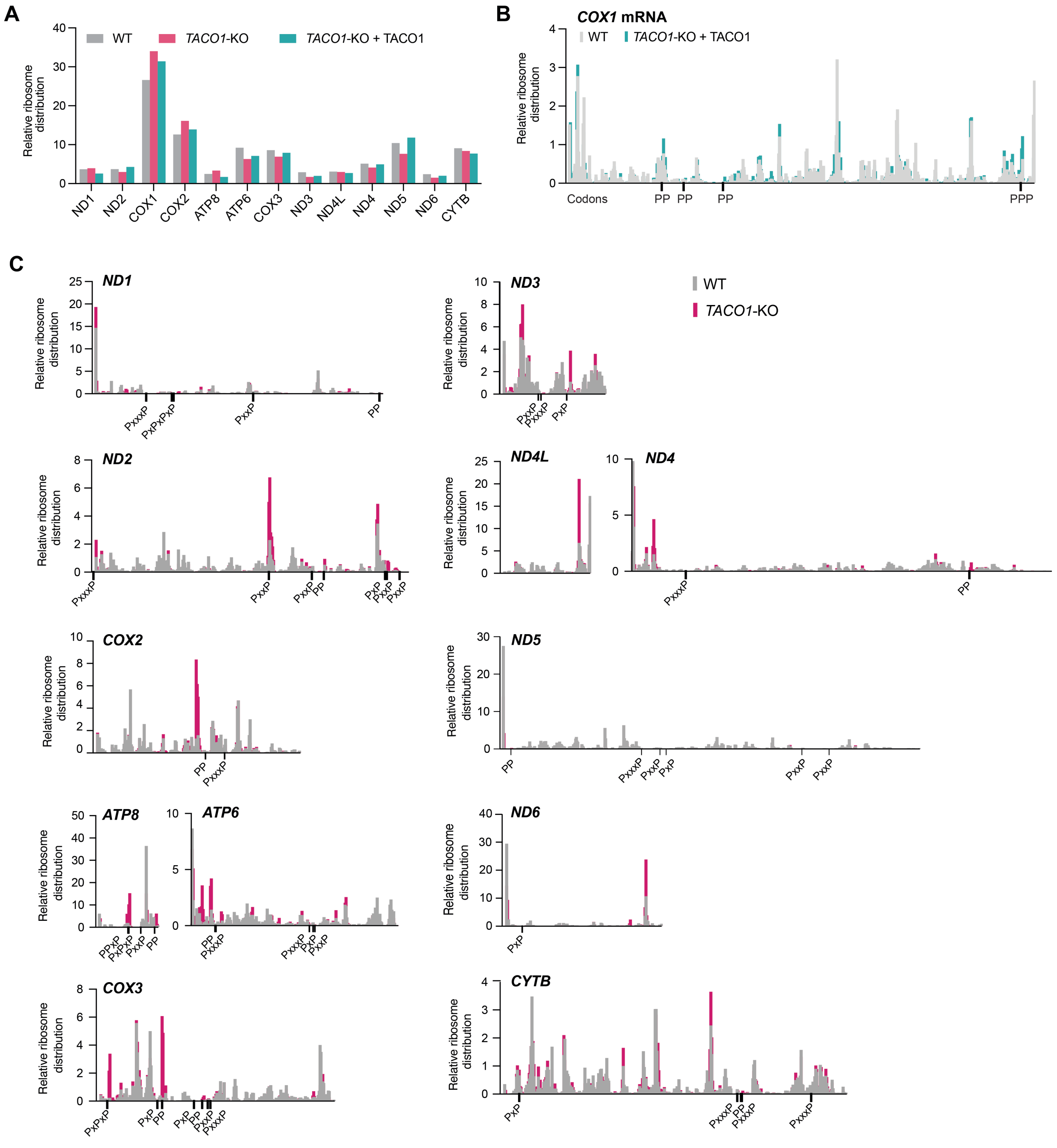

(A) Occupancy of mitoribosomes on each transcript relative to the total occupancy on all transcripts. **(B)** Relative occupancy of mitoribosomes on COX1 transcript in WT and TACO1 reconstituted cells. Read counts at each codon position were normalized to the total number of read counts for COX1. Proline-rich regions of more than two prolines per 5 amino acid sequence length are highlighted. **(C)** Relative occupancy of mitoribosomes on all mitochondrial transcripts in WT and *TACO1*-KO cells. Read counts at each codon position were normalized to the total number of read counts for each transcript. Proline-rich regions of more than two prolines per 5 amino acid sequence length are highlighted.

Supplementary Figure S4. Location of mtLSU proteins with higher specificity scores as preys in BioID assays using TACO1 as the bait. Related to Fig. 2

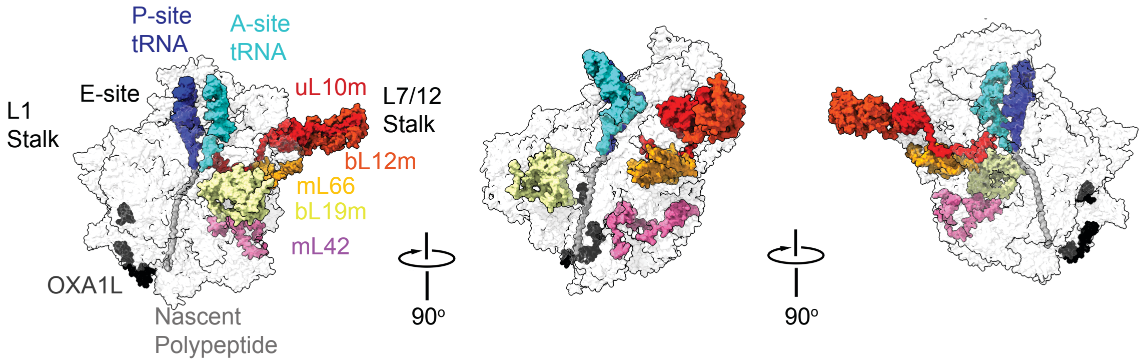

Cryo-EM structure of the human mitoribosome LSU in complex with OXA1L (PDB: 6zm5), highlighting the five proteins with higher specificity scores as preys in BioID assays using TACO1 as the bait. The figures were prepared using ChimeraX (1)

Supplementary Figure S5. Structural comparison of translation factors TACO1, EF-P, eIF5A, and ABC-F EttA. Related to Fig. 3.

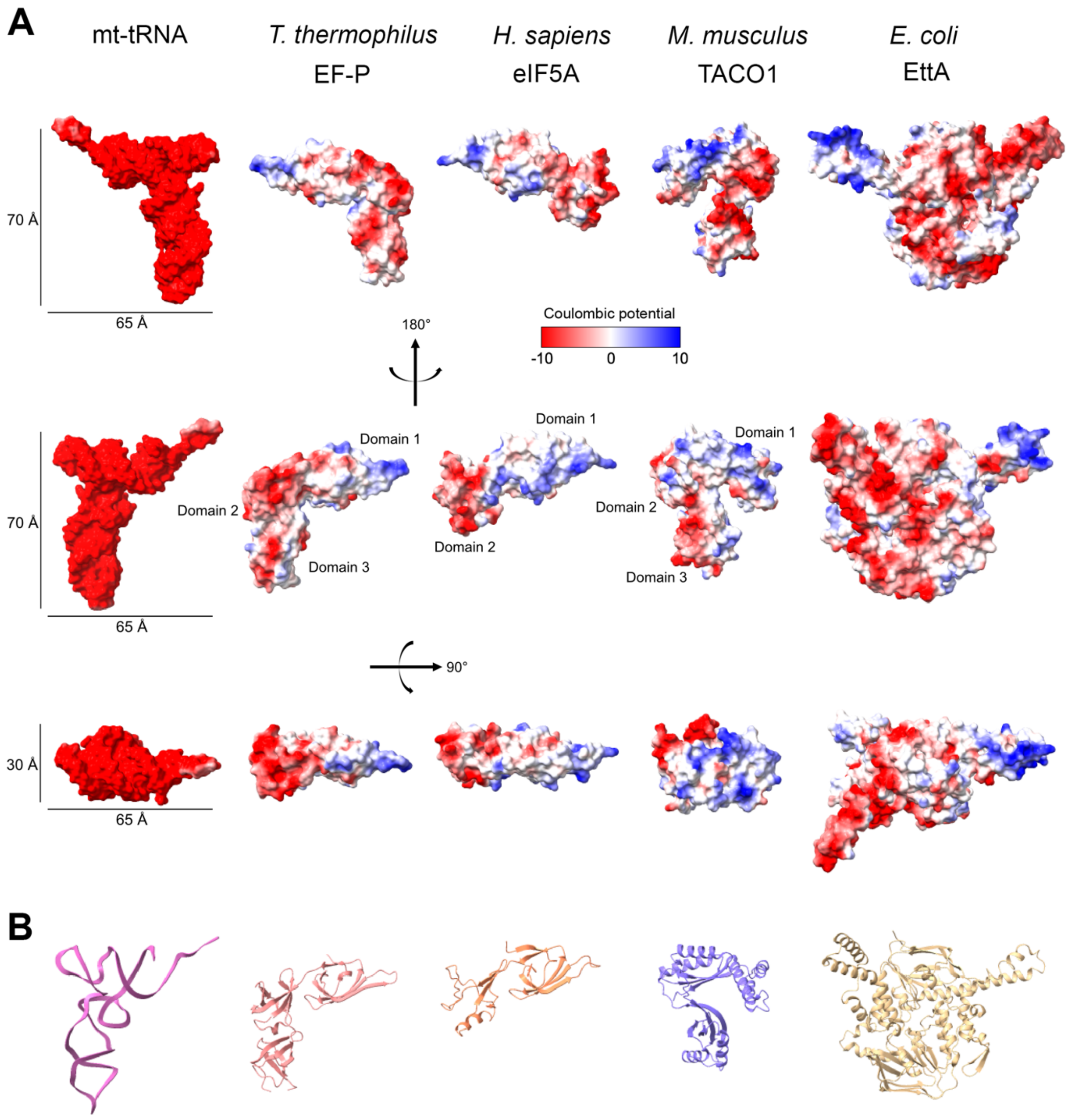

Structural comparison of cryo-EM structure of mt-tRNA (PDB ID: 6ZM5), crystal structure of *Thermus thermophilus* EF-P (PDB ID: 1UEB)(2), cryo-EM structure of *Homo sapiens* eIF5A (PDB ID: 8A0E)(3), crystal structure of *Mus musculus* TACO1 (PDB ID: 5EKZ) (4), and *Escherichia coli* ABC-F protein EttA (PDB ID: 3J5S)(5). (**A**) Electrostatic surface potentials. Colors display the electrostatic charges on the surface (red = negative, white = neutral, blue = positive). (**B**) Ribbon representation of the structures.

Supplementary Tables

Supplementary Table S1. Key Resources

REAGENT or RESOURCE	SOURCE	IDENTIFIER
Antibodies		
Mouse monoclonal anti MT-CO1	Abcam	Ab14705
Mouse monoclonal anti MT-CO2	Abcam	Ab110258
Mouse monoclonal anti beta-actin	Proteintech	60008-1-Ig
Mouse monoclonal anti ATP5A	Abcam	Ab14748
Rabbit polyclonal anti TACO1	Origene	TA331915
Rabbit polyclonal anti PTCD3 (ms39)	ThermoFisher	PA5-59455
Rabbit polyclonal anti MRPL14 (uL14m)	Abcam	Ab138281
Bacterial and virus strains		
NEB 5-alpha competent bacteria	NEB	C2987H
Chemicals, peptides, and recombinant proteins		
DMEM, high glucose, pyruvate	ThermoFisher	11995073
DMEM, high glucose, no glutamine, no methionine, no cystine	ThermoFisher	21013024
Freestyle™ 293 Expression Medium	ThermoFisher	12-338-026
DPBS, no calcium, no magnesium	ThermoFisher	14190144
Fetal bovine serum	Sigma	12303C
Uridine	Sigma	58968
GlutaMAX™ Supplement	ThermoFisher	35050061
Collagen I, Rat Tail	ThermoFisher	A1048301
Complete EDTA Free Protease Inhibitor Cocktail	Sigma	11873580001
EasyTag™ L-[^{35}S]-Methionine	Perkin-Elmer	NEG709A
Emetine dihydrochloride	ChemCruz	SC-202600
PMSF protease inhibitor	Thermo	36978
Digitonin, High Purity	Sigma	300410
Hygromycin B	Corning	45000-806
Puromycin dihydrochloride from Streptomyces alboniger	Sigma	P7255
Erythromycin	Sigma	E6376
Trizol	ThermoFisher	15596026
Critical commercial assays		
High-Capacity cDNA Reverse Transcription Kit	ThermoFisher	4368814
SsoAdvanced Universal SYBR Green Supermix	Bio-Rad	1725271
Q5 Site-directed Mutagenesis Kit	New England Biolabs	E0554S
Deposited data		
SILAC AP-MS data	PRIDE	PXD051090
Ribosome profiling data	GEO	https://drive.google.com/drive/folders/1teCn-iQmX-4zoCHuoTRFt5r2RmQdUIbX?usp=sharing.
Experimental models: Cell lines		
HEK293T	ATCC	CRL-3216
HEK293T *TACO1-KO*	This work	

HEK293T *TACO1-KO* + *TACO1*	This work	
HEK293T *TACO1-KO* + *TACO1-FLAG*	This work	
143B	ATCC	CRL-8303
COX1 cybrids (m.G6930A)	Gift from Dr. G. Manfredi (6)	
143B *TACO1-KO*	This work	
TACO1-KO COX1 cybrids (m.G6930A)	This work	
HEK293T + *pCMV6 EV*	This work	
HEK293T + *bL27m WT*	This work	
HEK293T + *bL27m K34A*	This work	
HEK293T + *bL27m S35A*	This work	
HEK293T + *bL27m S35F*	This work	
HEK293T + *bL27m S46F*	This work	
HEK293T *TACO1-KO* + *pCMV6 EV*	This work	
HEK293T *TACO1-KO* + *bL27m WT*	This work	
HEK293T *TACO1-KO* + *bL27m K34A*	This work	
HEK293T *TACO1-KO* + *bL27m S35A*	This work	
HEK293T *TACO1-KO* + *bL27m S35F*	This work	
HEK293T *TACO1-KO* + *bL27m S46F*	This work	
HEK293T *TACO1-OE*	This work	
HEK293T *TACO1-KO* + *TACO1-OE*	This work	
Oligonucleotides		
COX1_qPCR_F	This work	CTCTTCGTCTGATCCGTCCT
COX1_qPCR_R	This work	ATTCCGAAGCCTGGTAGGAT
COX2_qPCR_F	This work	ACGAGTACACCGACTACGGC
COX2_qPCR_R	This work	CGGGAATTGCATCTGTTTTT
COX3_qPCR_F	This work	CCCACCAATCACATGCCTAT
COX3_qPCR_R	This work	GTGGCCTTGGTATGTGCTTT
CYB_qPCR_F	This work	TGAAACTTCGGCTCACTCCT
CYB_qPCR_R	This work	AGAATATTGAGGCGCCATTG
ND4_qPCR_F	This work	GCCTACGACAAACAGACCTAAAAT
ND4_qPCR_R	This work	TTTGGATGAGAATGGCTGTTACTA
ND5_qPCR_F	This work	AAACAACCCAGCTCTCCCTAA
ND5_qPCR_R	This work	ATGAACAGTTGGAATAGGTT
12S_qPCR_F	This work	TAGAGGAGCCTGTTCTGTAATCGA
12S_qPCR_R	This work	TGCGCTTACTTTGTAGCCTTCAT
16S_qPCR_F	This work	AGAGAGTAAAAAATTTAACACCCAT
16S_qPCR_R	This work	TTCTATAGGGTGATAGATTGGTCC
ActinB primer cocktail for qPCR_Sybr Green	GeneCopoeia	HQP016381
Recombinant DNA		
TACO1 (NM_016360) Human Tagged ORF Clone	Origene	RC202948
MRPL27 (NM_016504) Human Untagged Clone	Origene	SC127560
Software and algorithms		

Adobe Illustrator 2023	v.27.4	https://www.adobe.com/products/illustrator.html
Fiji	v.2.15.0	https://imagej.net/software/fiji/
Gel Analyzer	v.23.1	http://www.gelanalyzer.com/?i=1
GraphPad Prism	v.10.0.3 (217)	https://www.graphpad.com
Other		
Kodak X-OMAT X-ray film	Sigma	F1274-50EA
eBlot L1 Protein Transfer System	Genscript	L00686
NativePAGE™ 3 to 12%, Bis-Tris Mini Protein Gels	ThermoFisher	BN1001BOX

Supplementary Table S4. Mitoribosome stalling effect of PP motifs and TACO1-mediated alleviation. RRD, mRNA-specific relative ribosome distribution in mitoribosome profile assays. Pausing scores reported in studies *in vitro* and in bacteria were used as a reference (7-10). * Denotes end of translation (final amino acid). Prolines are in red, and proline-flanking amino acids are in blue.

PROTEIN	MOTIF	MOTIF PAUSING SCORE (7)		STALLING IN *TACO1*-KO (RRD)	ALLEVIATED BY TACO1 (RRD)
		(X)PP	PP(X)		
COX1	104-LLPPSL	2.0	30.0	Yes (4.0)	Yes (0.6)
COX1	128-VYPPLA	3.5	6.0	No	-
COX1	171-MKPPAM	3.0	12.0	Yes (0.5)	Yes (0.0)
COX1	497-GCPPPY	50.0	50.0	Yes (4.0)	Yes (0.5)
COX2	121-MLPPLF	2.0	6.0	No	-
COX3	71- HTPPVQ	2.0	13.0	Yes (6)	Yes (0.5)
COX3	115-HWPPTG	2.0	13.0	No	-
ND1	313-SIPPQT*	3.5	10.0	No	-
ND2	253-GLPPLT	2.0	6.0	No	-
ND4	368-ALPPTI	2.0	13.0	No	-
ND5	15-LIPPIL	3.0	7.0	No	-
ATP8	34-HLPPSPKPMK	2.0	30.0	Yes (15.0)	Yes (1.0)
ATP8	63-SLPPQS*	2.0	20.0	No	-
ATP6	25-LFPPLLIPTS	2.0	6.0	No	-
CYTB	263-NTPPHIKPEW	1.2	10.0	No	-

Supplementary Table S5. Mitoribosome stalling effect of non-consecutive P-containing motifs and TACO1-mediated alleviation. RRD, mRNA-specific relative ribosome distribution in mitoribosome profiling. Prolines are in red, and proline-flanking amino acids are in blue.

PROTEIN	MOTIF	STALLING IN *TACO1*-KO (RRD)	ALLEVIATED BY TACO1 (RRD)
COX3	11-VKPSPWPLT	Yes (3.5)	Yes (0.0)
ND1	175-LLPSWPLA	No	-
ND5	228-LHPWLPSA	No	-
ND3	73-LLPLPWA	No	-
ATP8	48-NKPWEPKW	No	-
ND2	193-VLPYNPNM	Yes (6.5)	Yes (2.1)
ND1	87-WTPLPMPNPLV	No	-
ND2	241-LTPLIPST	No	-
ND2	336-LLPISPFM	No	-
ND5	472-ISPASPFQ	No	-
ND2	319-TKPTPFLPTL	No	-
ND3	40-FDPMSPARVPFS	No	-
ATP6	132-GTPTPLIPML	No	-

Supplementary Table S6. Mitoribosome stalling effect of motifs that are not proline rich. RRD, mRNA-specific relative ribosome distribution in mitoribosome profile assays. * Denotes end of translation (final amino acid). Prolines are in red, proline-flanking amino acids are in blue, and amino acids where the pausing occurred are in purple.

PROTEIN	MOTIF	STALLING IN *TACO1*-KO (RRD)	ALLEVIATED BY TACO1 (RRD)
COX2	112-DYGGLIFNSYMLPPLF	Yes (8.0)	Yes (1.8)
ND4L	84-TYGLDYVHNLNLLQC*	Yes (21.0)	Yes (6.0)
ND3	73-LLPLPWALQTTNL	Yes (3.9)	Yes (1.0)
ND6	151-WLVVVTGW	Yes (25.0)	Yes (10.0)
ATP6	5-LFASFIAPT	Yes (4.0)	Yes (0.5)
ATP6	16-GLPAAVL	Yes (4.5)	Yes (1.0)

Additional Supplementary Data

Presented in separate file:

Supplementary data file SD1: Alignment of the mitochondrial proteomes of human (*Homo sapiens*), mouse (*Mus musculus*), zebrafish (Danio rerio), frog (*Xenopus laevis*), fruit fly (*Drosophila melanogaster*) and budding yeast (*Saccharomyces cerevisiae*). Prolines are highlighted in red.

Additional Supplementary Tables

Presented in separate files:

Supplementary Table S2. List of TACO1 interactors obtained by applying the SILAC AP-MS approach. Related to Fig. 3.

Supplementary Table S3. Catalog of high-confidence proximity partners for TACO1 obtained by applying the BioID approach. Related to Fig. 3.

References

1. Pettersen, E.F., Goddard, T.D., Huang, C.C., Meng, E.C., Couch, G.S., Croll, T.I., Morris, J.H. and Ferrin, T.E. (2021) UCSF ChimeraX: Structure visualization for researchers, educators, and developers. *Protein Sci.*, **30**, 70-82.
2. Hanawa-Suetsugu, K., Sekine, S., Sakai, H., Hori-Takemoto, C., Terada, T., Unzai, S., Tame, J.R., Kuramitsu, S., Shirouzu, M. and Yokoyama, S. (2004) Crystal structure of elongation factor P from *Thermus thermophilus* HB8. *Proc. Natl. Acad. Sci. U. S. A.*, **101**, 9595-9600.
3. Wątor, E., Wilk, P., Biela, A., Rawski, M., Zak, K.M., Steinchen, W., Bange, G., Glatt, S. and Grudnik, P. (2023) Cryo-EM structure of human eIF5A-DHS complex reveals the molecular basis of hypusination-associated neurodegenerative disorders. *Nat. Commun.*, **14**, 1698.
4. Richman, T.R., Spahr, H., Ermer, J.A., Davies, S.M., Viola, H.M., Bates, K.A., Papadimitriou, J., Hool, L.C., Rodger, J., Larsson, N.G. *et al.* (2016) Loss of the RNA-binding protein TACO1 causes late-onset mitochondrial dysfunction in mice. *Nat Commun.*, **7**, 11884.
5. Chen, B., Boël, G., Hashem, Y., Ning, W., Fei, J., Wang, C., Gonzalez, R.L., Jr., Hunt, J.F. and Frank, J. (2014) EttA regulates translation by binding the ribosomal E site and restricting ribosome-tRNA dynamics. *Nat. Struct. Mol. Biol.*, **21**, 152-159.
6. D'Aurelio, M., Pallotti, F., Barrientos, A., Gajewski, C.D., Kwong, J.Q., Bruno, C., Beal, M.F. and Manfredi, G. (2001) In vivo regulation of oxidative phosphorylation in cells harboring a stop-codon mutation in mitochondrial DNA-encoded cytochrome c oxidase subunit I. *J. Biol. Chem.*, **276**, 46925-46932.
7. Woolstenhulme, C.J., Guydosh, N.R., Green, R. and Buskirk, A.R. (2015) High-precision analysis of translational pausing by ribosome profiling in bacteria lacking EFP. *Cell Rep.*, **11**, 13-21.

8. Woolstenhulme, C.J., Parajuli, S., Healey, D.W., Valverde, D.P., Petersen, E.N., Starosta, A.L., Guydosh, N.R., Johnson, W.E., Wilson, D.N. and Buskirk, A.R. (2013) Nascent peptides that block protein synthesis in bacteria. *Proc. Natl. Acad. Sci. U. S. A.*, **110**, E878-887.
9. Seip, B., Sacheau, G., Dupuy, D. and Innis, C.A. (2018) Ribosomal stalling landscapes revealed by high-throughput inverse toeprinting of mRNA libraries. *Life Sci. Alliance*, **1**, e201800148.
10. Peil, L., Starosta, A.L., Lassak, J., Atkinson, G.C., Virumäe, K., Spitzer, M., Tenson, T., Jung, K., Remme, J. and Wilson, D.N. (2013) Distinct XPPX sequence motifs induce ribosome stalling, which is rescued by the translation elongation factor EF-P. *Proc. Natl. Acad. Sci. U. S. A.*, **110**, 15265-15270.
