# Supplementary material for: The human mitochondrial translation factor TACO1 alleviates mitoribosome stalling at polyproline stretches
Source: Nucleic Acids Res. 2024 Jul 22;52(16):9710–26. doi: 10.1093/nar/gkae645 (PMC11381339; doi:10.1093/nar/gkae645)
Supplement: gkae645_Supplemental_Files [file gkae645_supplemental_files.zip › TACO1-Supplementary_data_file_SD1.pdf]

Supplemental Data 1 (SD1).

Alignment of the mitochondrial proteomes of human (*Homo sapiens*), mouse (*Mus musculus*), zebrafish (*Danio rerio*), frog (*Xenopus laevis*), fruit fly (*Drosophila melanogaster*) and budding yeast (*Saccharomyces cerevisiae*). Prolines are highlighted in red.

ND1

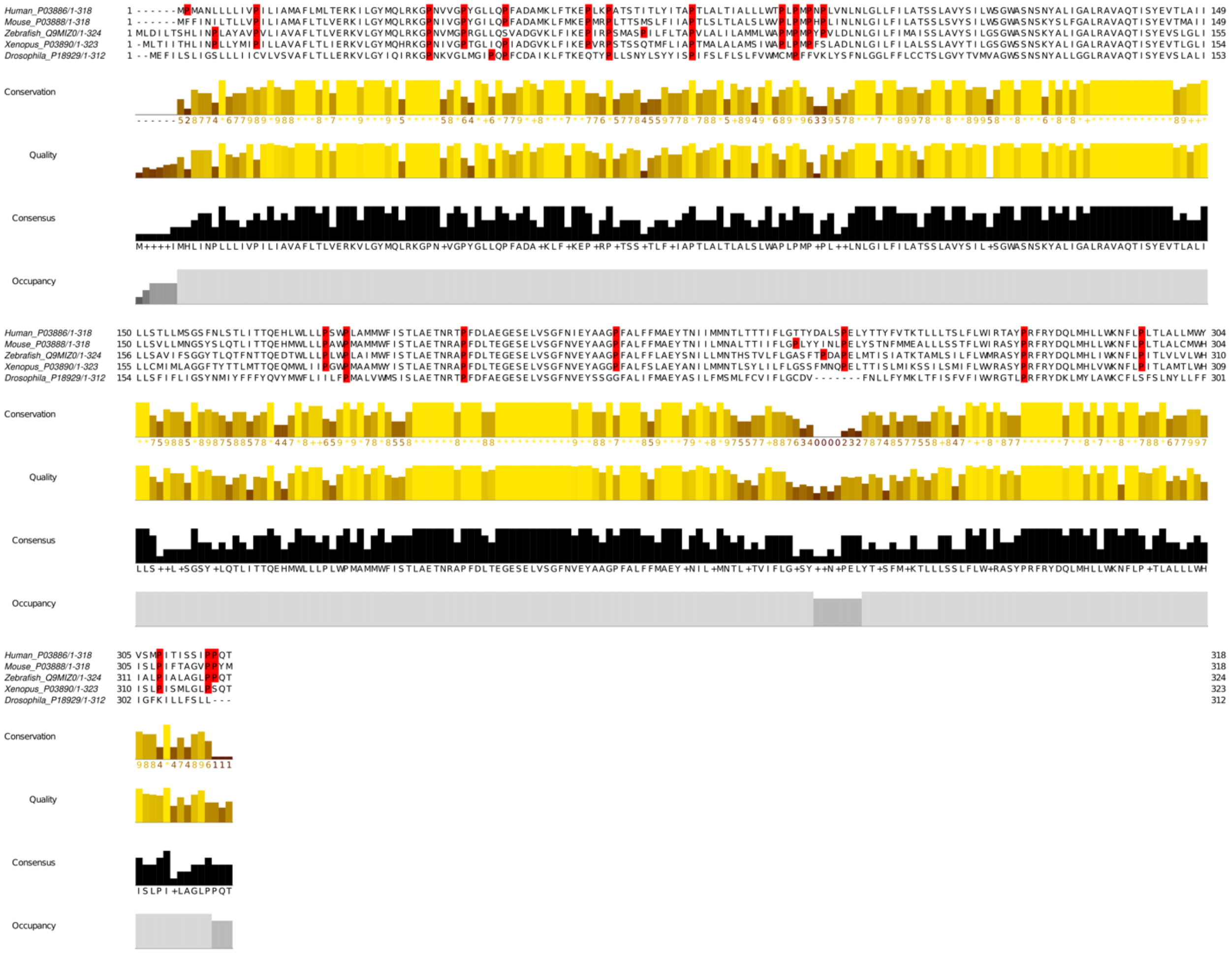

ND2

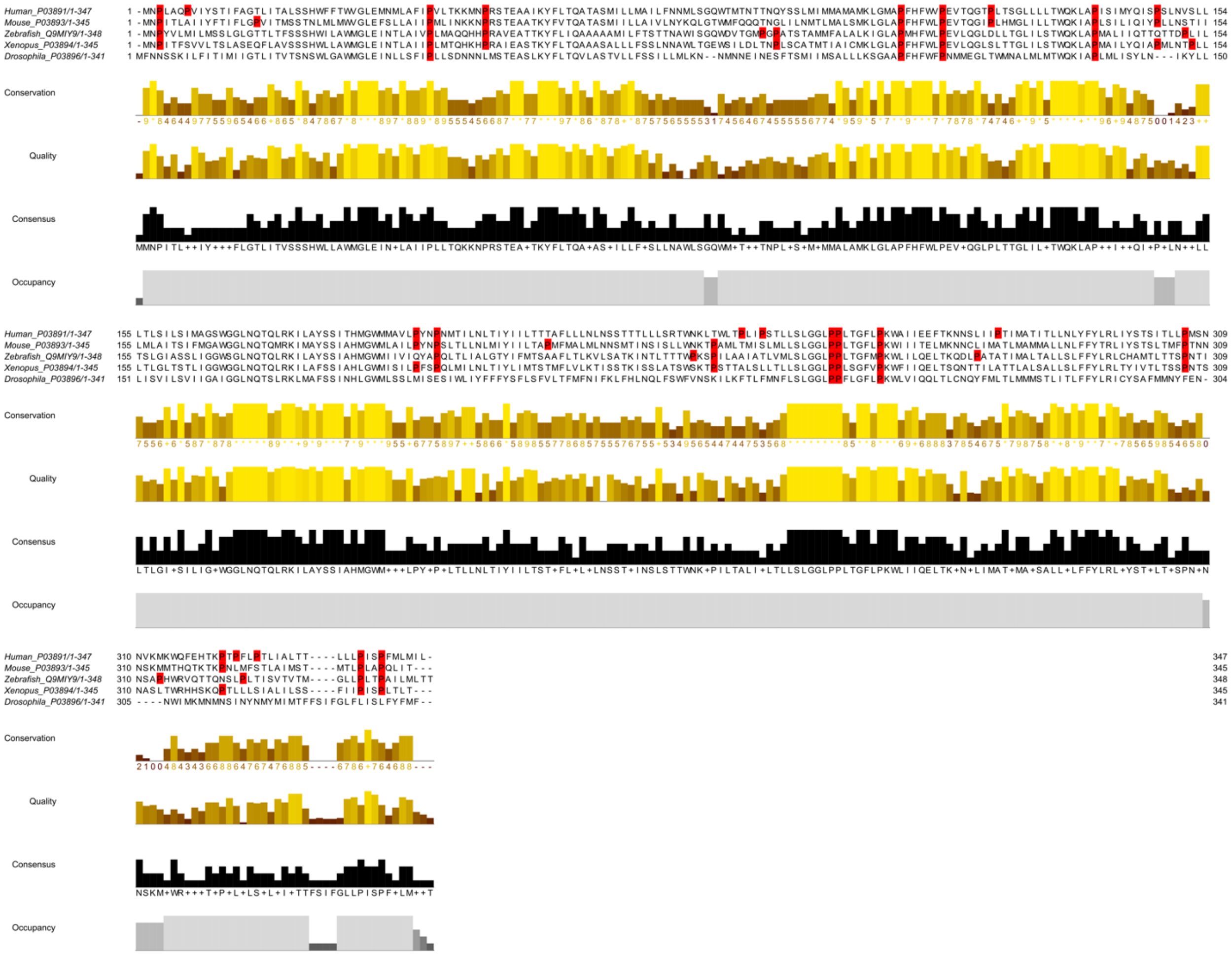
Human_P03891/1-347
Mouse_P03893/1-345
Zebrafish_Q9MIY9/1-348
Xenopus_P03894/1-345
Drosophila_P03896/1-341
Conservation
Quality
Consensus
Occupancy

ND3

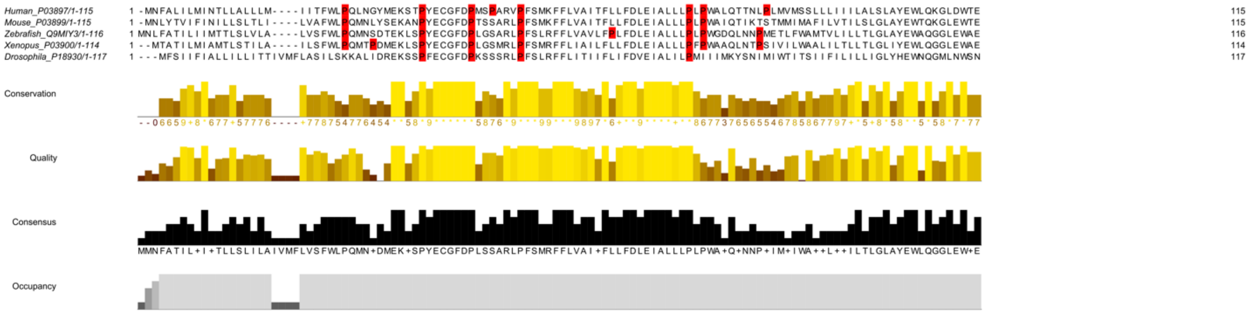
Human_P03897/1-115
Mouse_P03899/1-115
Zebrafish_Q9MIY3/1-116
Xenopus_P03900/1-114
Drosophila_P18930/1-117
115
115
116
114
117
Conservation
Quality
Consensus
Occupancy

ND4

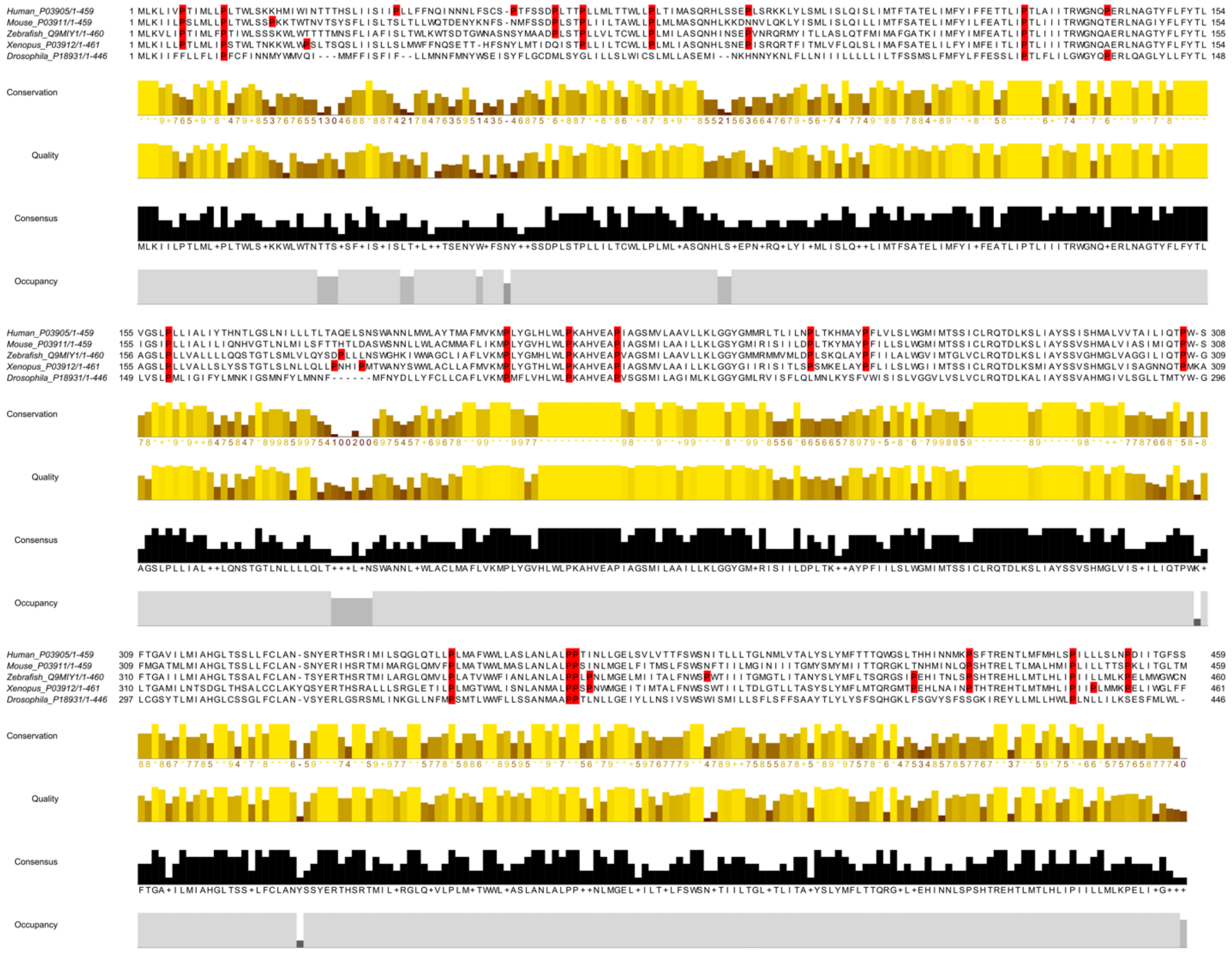
Human_P03905/1-459
Mouse_P03911/1-459
Zebrafish_Q9MIY1/1-460
Xenopus_P03912/1-461
Drosophila_P18931/1-446
Conservation
Quality
Consensus
Occupancy

ND4L

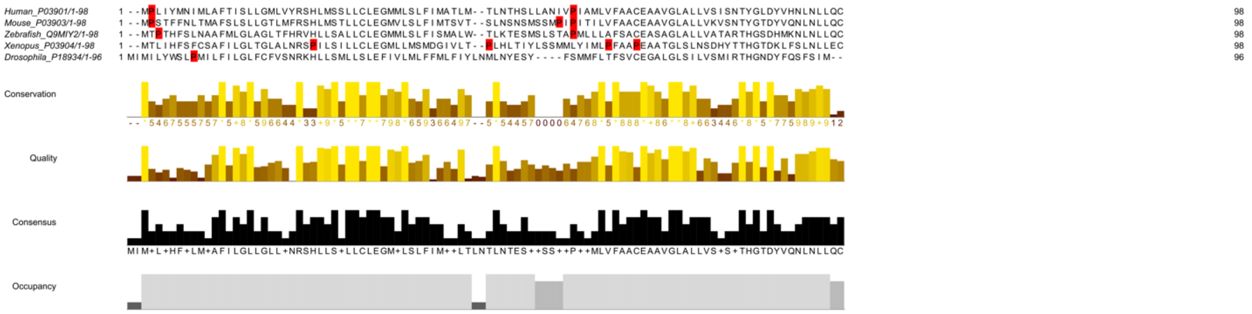

Human_P03901/1-98 1 --MPLIYMNIMLAFTISLLGMLVYRSHLMSSLLCLEGMMLSLFIMATLM--TLNTHSLLANIVPIAMLVFAACEAAVGLALLVSISNTYGLDYVHNLNLLQC 98
Mouse_P03903/1-98 1 --MPSTFFNLTMAFSLSLLGTLMFRSHLMSTLLCLEGMVLSLFIMTSVT--SLNSNSMSSMPIPITILVFAACEAAVGLALLVKVSNTYGTDYVQNLNLLQC 98
Zebrafish_Q9MIY2/1-98 1 --MTPTHFSLNAAFMLGLAGLTFHRVHLLSALLCLEGMMLSLFISMALW--TLKTESMSLSTAPMLLLAFSACEASAGLALLVATARTHGSDHMKNLNLLQC 98
Xenopus_P03904/1-98 1 --MTLIHFSFCSAFILGLTGLALNRSPILSILLCLEGMLLMSMDGIVLT--PLHLTIYLSSMMLYIMLPFAAPEAATGLSLNSDHYTTHGTDKLFSLNLLEC 98
Drosophila_P18934/1-96 1 MIMILYWSLPMILFILGLFCFVSNRKHLLSMLLSLEFIVLMLFFMLFIYLNMLNYESY----FSMMFLTFSVCEGALGLSILVSMIRTHGNDYFQSFSIM-- 96
Conservation
--*5467555757*5+8*596644*33+9*5**7**798*659366497--5*54457000064768*5*888*+86**8+663446*8*5*775989+912
Quality
Consensus
MIM+L+HF+LM+AFILGLLGLL+NRSHLLS+LLCLEGM+LSLFIM++LTLNTLNTES++SS++P++MLVFAACEAAVGLALLVS+S+THGTDYVQNLNLLQC
Occupancy

ND5

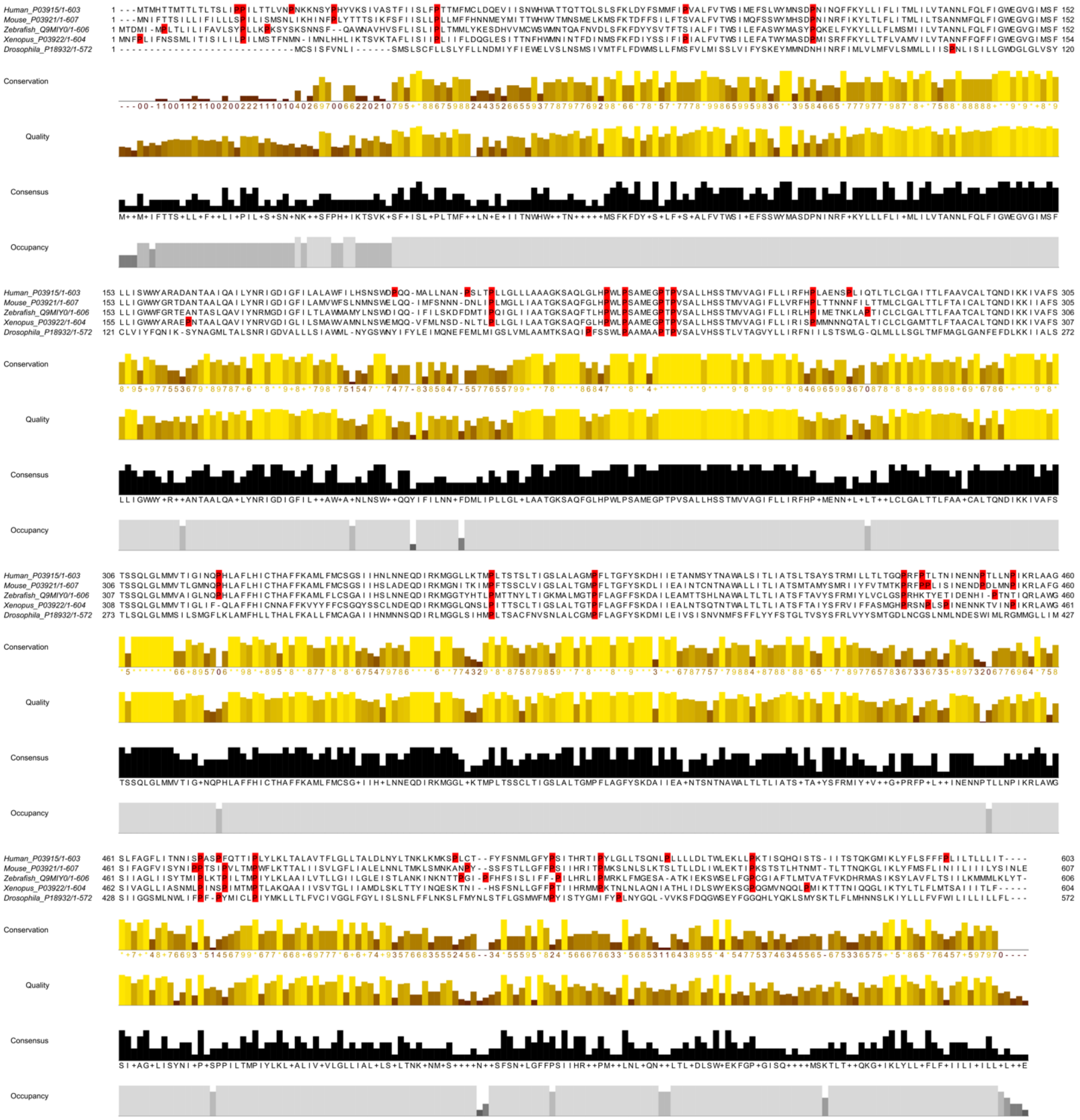
Human_P03915/1-603
Mouse_P03921/1-607
Zebrafish_Q9MIY0/1-606
Xenopus_P03922/1-604
Drosophila_P18932/1-572
Conservation
Quality
Consensus
Occupancy

ND6

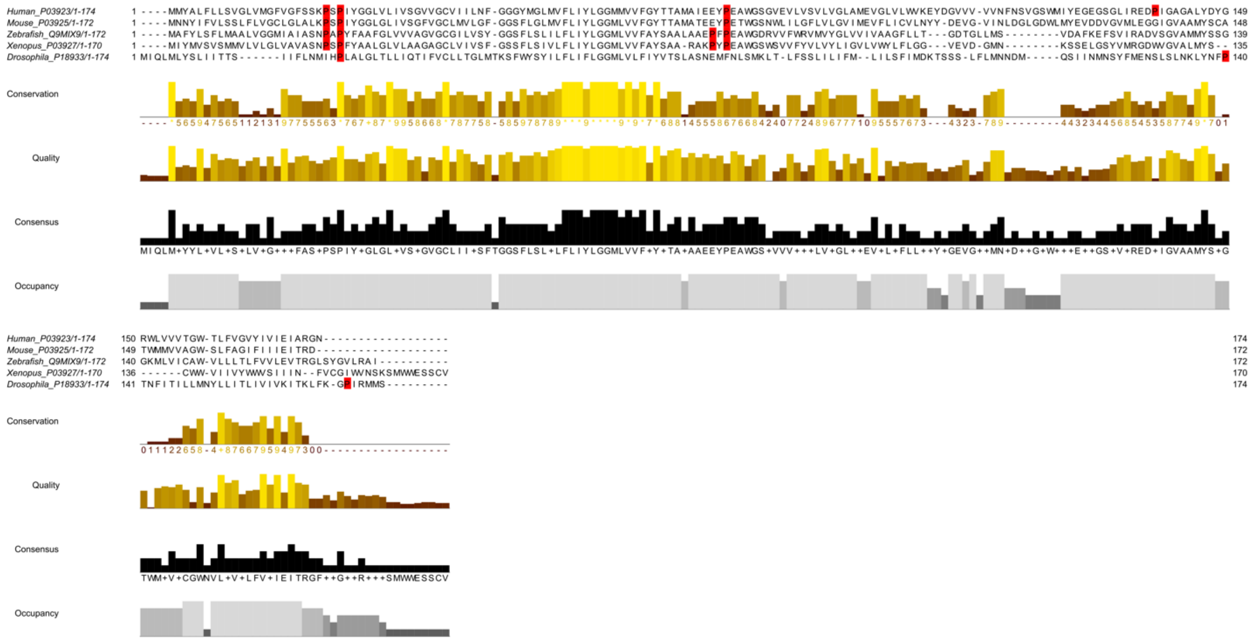
Human_P03923/1-174
Mouse_P03925/1-172
Zebrafish_Q9MIX9/1-172
Xenopus_P03927/1-170
Drosophila_P18933/1-174
Conservation
Quality
Consensus
Occupancy

CYB

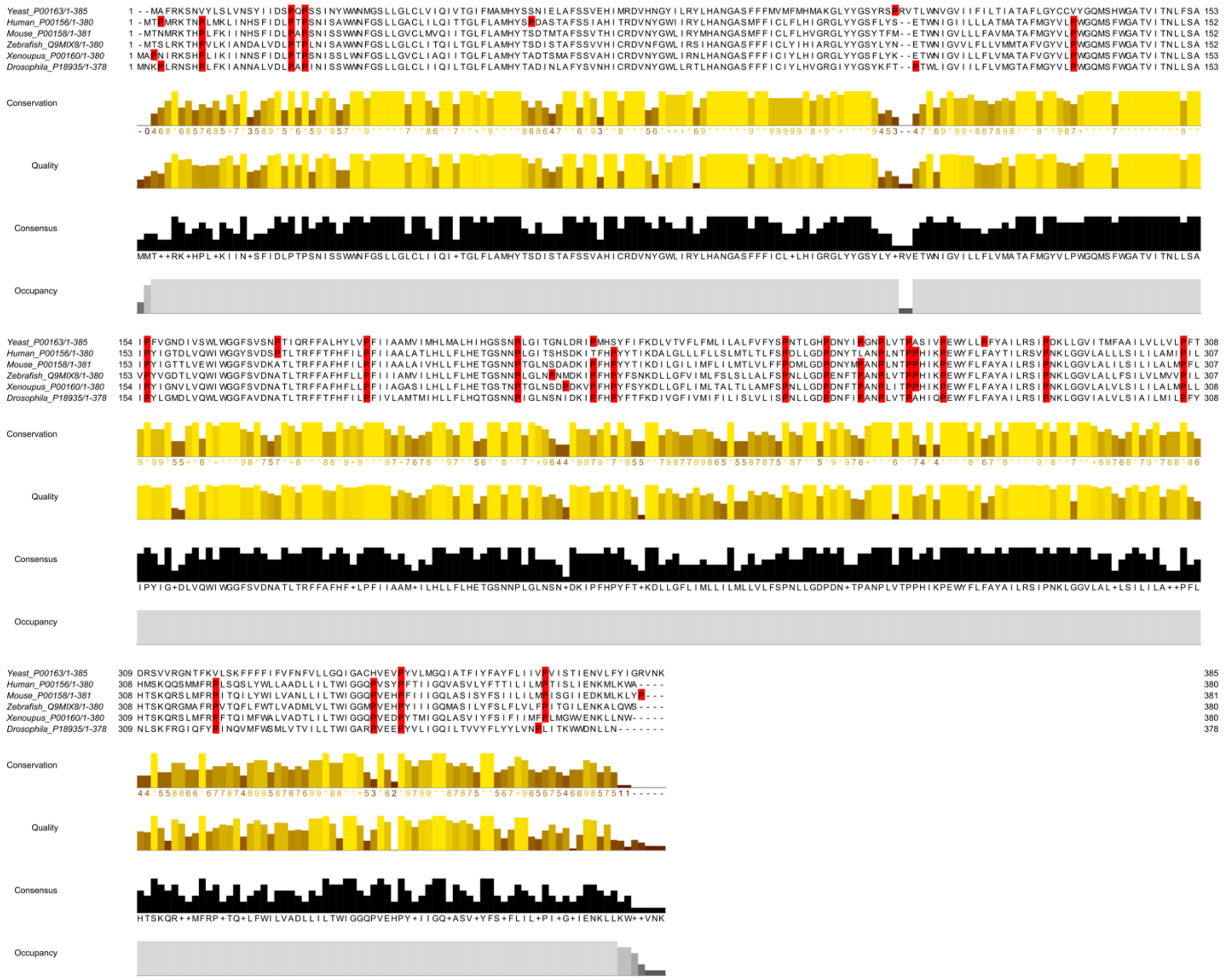
Yeast_P00163/1-385
Human_P00156/1-380
Mouse_P00158/1-381
Zebrafish_Q9MIX8/1-380
Xenoupus_P00160/1-380
Drosophila_P18935/1-378
Conservation
Quality
Consensus
Occupancy

COX1

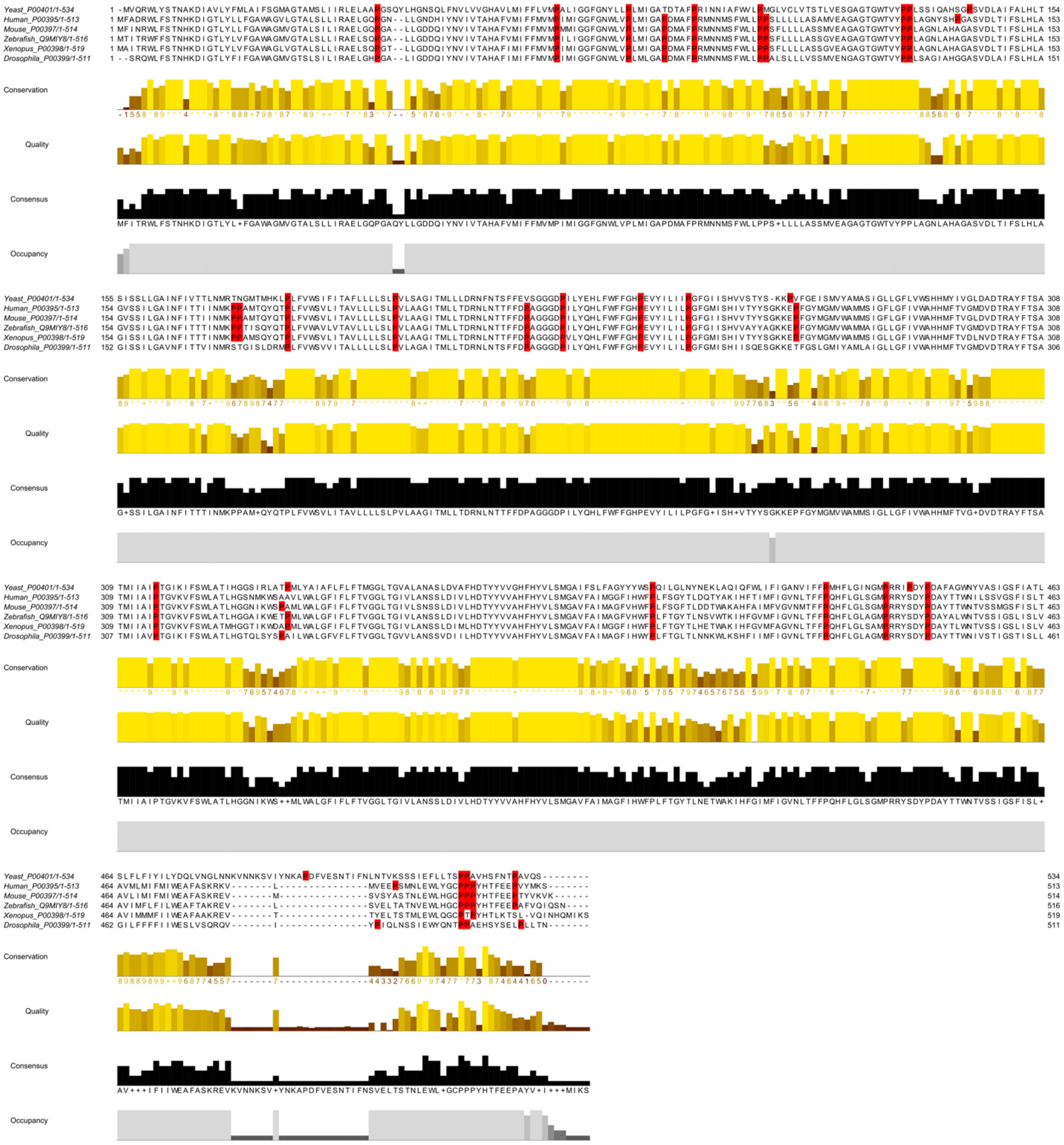
Yeast_P00401/1-534
Human_P00395/1-513
Mouse_P00397/1-514
Zebrafish_Q9MIY8/1-516
Xenopus_P00398/1-519
Drosophila_P00399/1-511
Conservation
Quality
Consensus
Occupancy

COX2

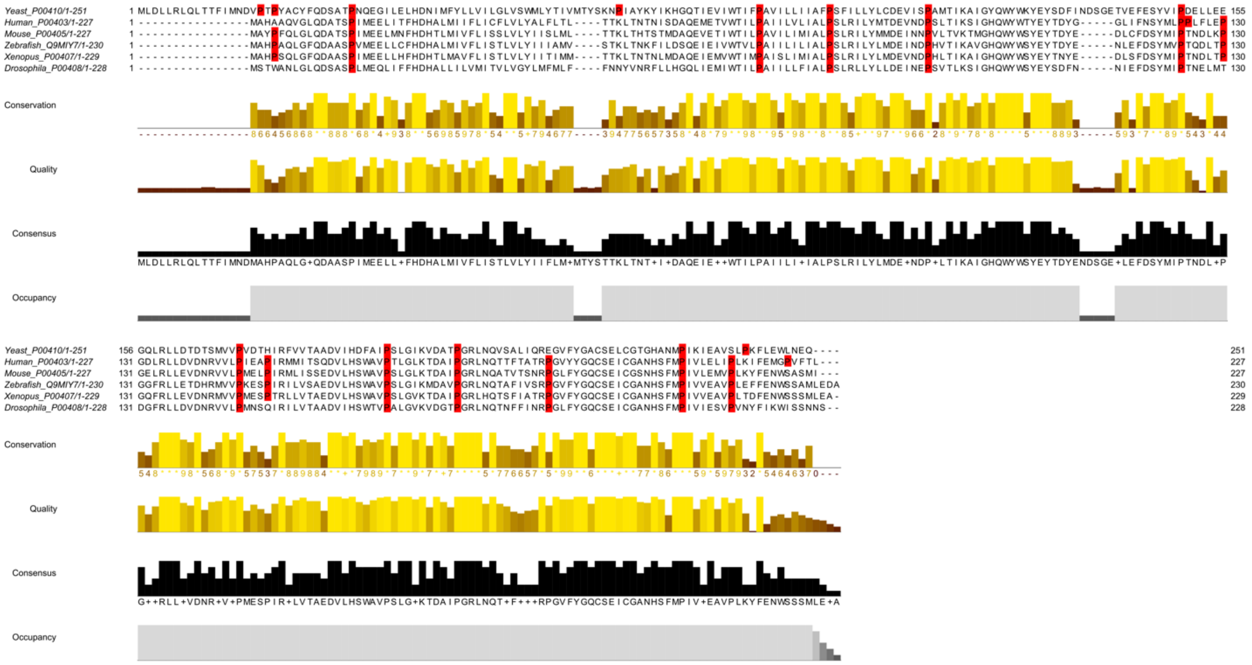
Yeast_P00410/1-251
Human_P00403/1-227
Mouse_P00405/1-227
Zebrafish_Q9MIY7/1-230
Xenopus_P00407/1-229
Drosophila_P00408/1-228
Conservation
Quality
Consensus
Occupancy

COX3

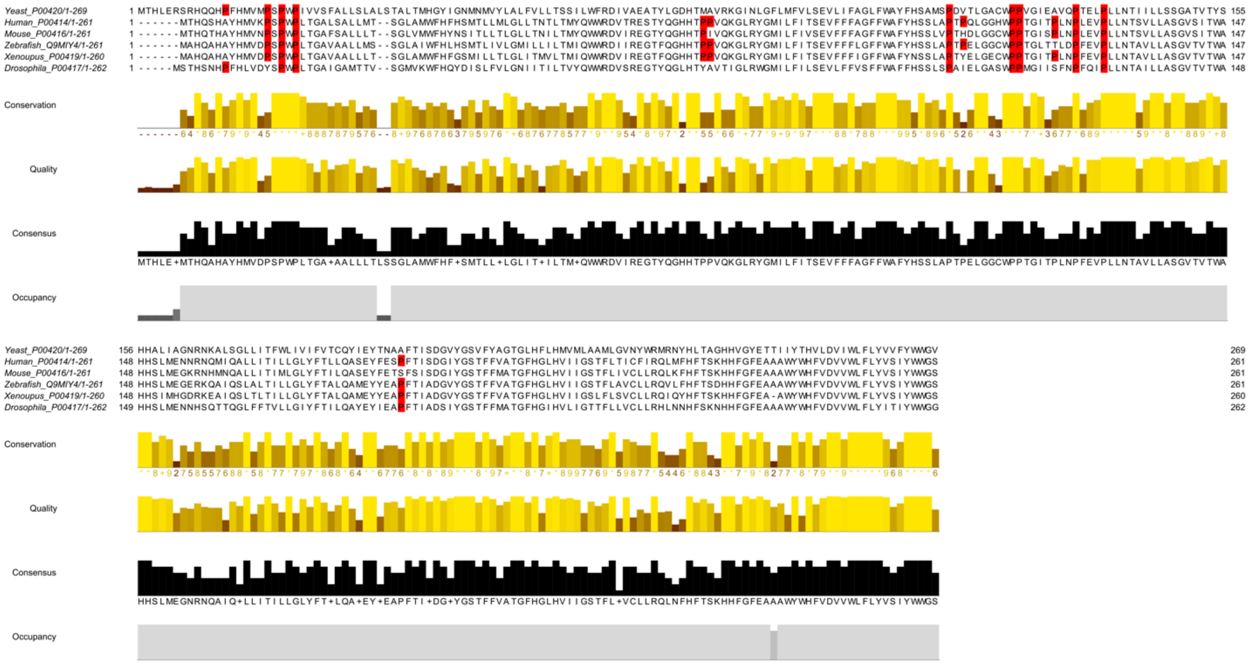
Yeast_P00420/1-269
Human_P00414/1-261
Mouse_P00416/1-261
Zebrafish_Q9MIY4/1-261
Xenoupus_P00419/1-260
Drosophila_P00417/1-262
Conservation
Quality
Consensus
Occupancy

ATP6

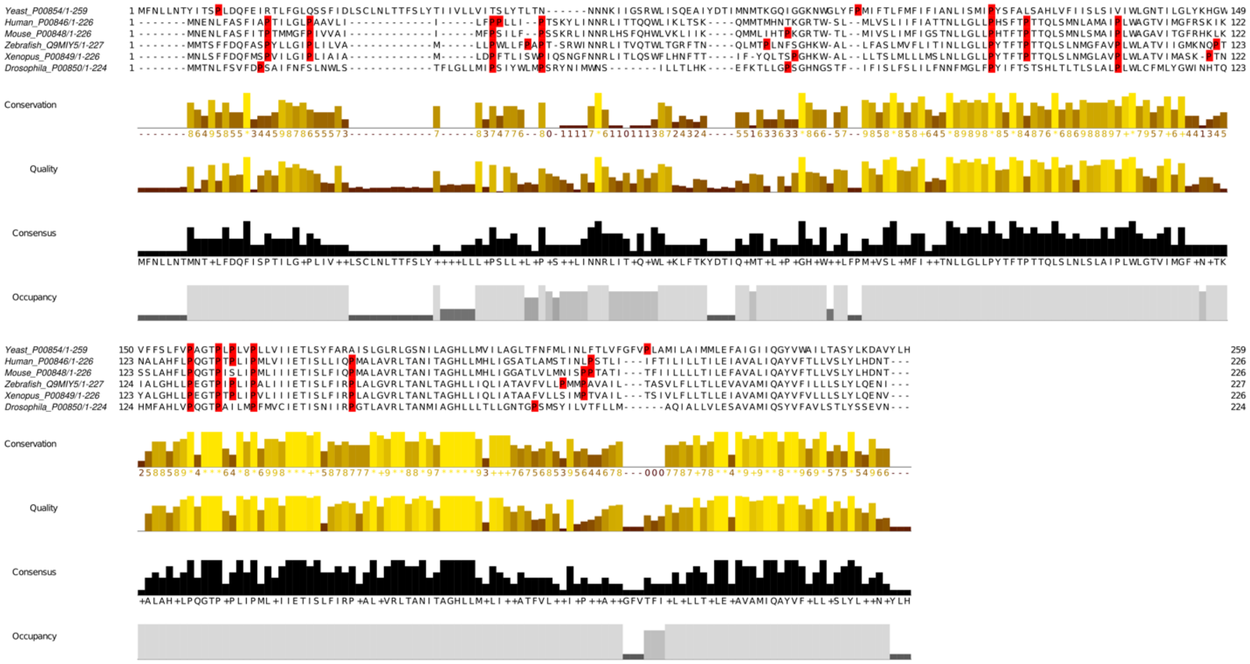

Yeast_P00854/1-259
Human_P00846/1-226
Mouse_P00848/1-226
Zebrafish_Q9MIY5/1-227
Xenopus_P00849/1-226
Drosophila_P00850/1-224
Conservation
Quality
Consensus
Occupancy

ATP8

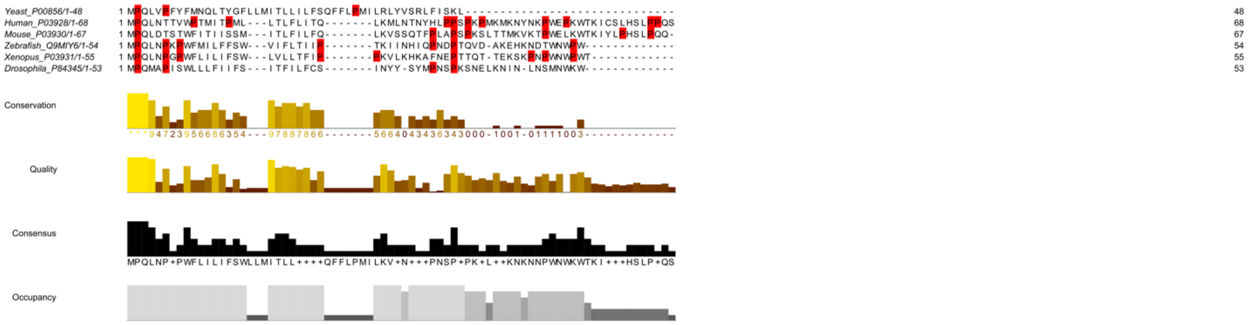
Yeast_P00856/1-48 1 MPQLVPFYFMNQLTYGFLLMITLLILFSQFFLPMILRLYVSRLFISKL------------------------ 48
Human_P03928/1-68 1 MPQLNTTVWPTMITPML---LTLFLITQ-------LKMLNTNYHLPPSPKPMKMKNYNKPWEPKWTKICSLHSLPPQS 68
Mouse_P03930/1-67 1 MPQLDTSTWFITIISSM---ITLFILFQ-------LKVSSQTFPLAPSPKSLTTMKVKTPWELKWTKIYLPHSLPQQ- 67
Zebrafish_Q9MIY6/1-54 1 MPQLNPKPWFMILFFSW---VIFLTIIP-------TKIINHIQPNDPTQVD-AKEHKNDTWNWPW------------ 54
Xenopus_P03931/1-55 1 MPQLNPGPWFLILIFSW---LVLLTFIP-------PKVLKHKAFNEPTTQT-TEKSKPNPWNWPWT----------- 55
Drosophila_P84345/1-53 1 MPQMAPISWLLLFIIFS---ITFILFCS-------INYY-SYMPNSPKSNELKNIN-LNSMNWKW------------ 53
Conservation
***94723956686354---97887866-------5664043436343000-1001-01111003-------------
Quality
Consensus
MPQLNP+PWFLILIFSWLLMITLL++++QFFLPMILKV+N+++PNSP+PK+L++KNKNNPWNWKWTKI+++HSLP+QS
Occupancy

ATP9

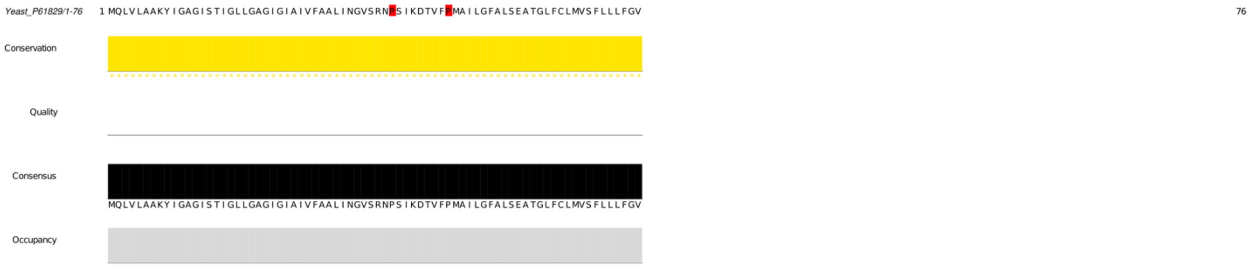
Yeast_P61829/1-76
1 MQLVLAAKYIGAGISTIGLLGAGIGIAIVFAALINGVSRNPSIKDTVFPMAILGFALSEATGLFCLMVSFLLLFGV
76
Conservation
Quality
Consensus
MQLVLAAKYIGAGISTIGLLGAGIGIAIVFAALINGVSRNPSIKDTVFPMAILGFALSEATGLFCLMVSFLLLFGV
Occupancy

VAR1

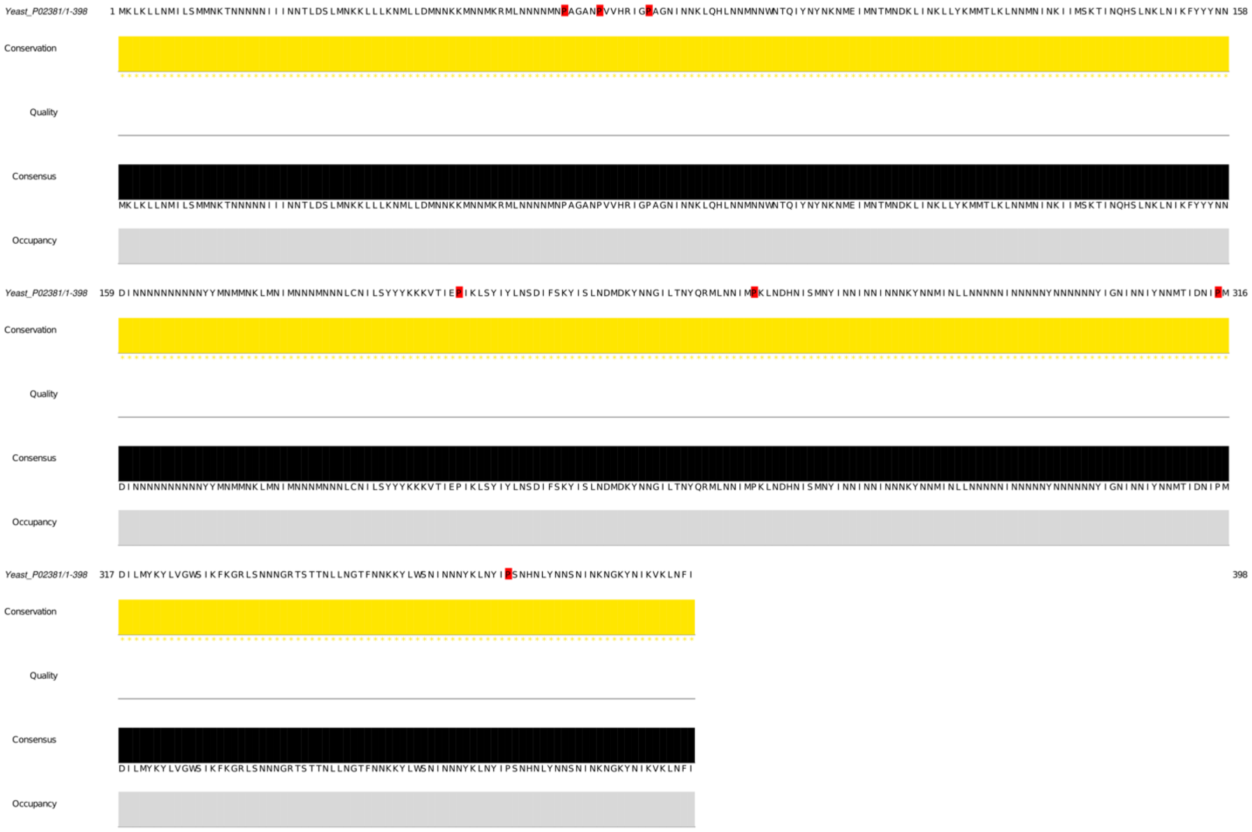
Yeast_P02381/1-398 1 MKLKLLNMILSMMNKTNNNNNIIINNTLDSLMNKKLLLKNMLLDMNNKKMNNMKRMLNNNNMNPAGANPVVHRIGPAGNINNKLQHLNNMNNWNTQIYNYNKNMEIMNTMNDKLINKLLYKMMTLKLNNMNINKIIMSKTINQHSLNKLNIKFYYYNN 158
Conservation
Quality
Consensus
MKLKLLNMILSMMNKTNNNNNIIINNTLDSLMNKKLLLKNMLLDMNNKKMNNMKRMLNNNNMNPAGANPVVHRIGPAGNINNKLQHLNNMNNWNTQIYNYNKNMEIMNTMNDKLINKLLYKMMTLKLNNMNINKIIMSKTINQHSLNKLNIKFYYYNN
Occupancy
Yeast_P02381/1-398 159 DINNNNNNNNNNYYMNMMNKLMNIMNNNMNNNLCNILSYYYKKKVTIEPIKLSYIYLNSDIFSKYISLNDMDKYNNGILTNYQRMLNNIMPKLNDHNISMNYINNINNINNNKYNNMINLLNNNNNINNNNNYNNNNNNYIGNINNIYNNMTIDNIPM 316
Conservation
Quality
Consensus
DINNNNNNNNNNYYMNMMNKLMNIMNNNMNNNLCNILSYYYKKKVTIEPIKLSYIYLNSDIFSKYISLNDMDKYNNGILTNYQRMLNNIMPKLNDHNISMNYINNINNINNNKYNNMINLLNNNNNINNNNNYNNNNNNYIGNINNIYNNMTIDNIPM
Occupancy
Yeast_P02381/1-398 317 DILMYKYLVGWSIKFKGRLSNNNGRTSTTNLLNGTFNNKKYLWSNINNNYKLNYIPSNHNLYNNSNINKNGKYNIKVKLNFI 398
Conservation
Quality
Consensus
DILMYKYLVGWSIKFKGRLSNNNGRTSTTNLLNGTFNNKKYLWSNINNNYKLNYIPSNHNLYNNSNINKNGKYNIKVKLNFI
Occupancy
